# Antimicrobial Proteins and Peptides in Avian Eggshell: Structural Diversity and Potential Roles in Biomineralization

**DOI:** 10.3389/fimmu.2022.946428

**Published:** 2022-07-27

**Authors:** Thierry Moreau, Joël Gautron, Maxwell T. Hincke, Philippe Monget, Sophie Réhault-Godbert, Nicolas Guyot

**Affiliations:** ^1^ INRAE, Université de Tours, BOA, Nouzilly, France; ^2^ Department of Innovation in Medical Education, Faculty of Medicine, University of Ottawa, Ottawa, ON, Canada; ^3^ Department of Cellular and Molecular Medicine, Faculty of Medicine, University of Ottawa, Ottawa, ON, Canada; ^4^ INRAE, CNRS, IFCE, Université de Tours, PRC, Nouzilly, France

**Keywords:** avian egg, eggshell, calcite, antimicrobial peptides and proteins, biomineralizing properties, 3D protein structure

## Abstract

The calcitic avian eggshell provides physical protection for the embryo during its development, but also regulates water and gaseous exchange, and is a calcium source for bone mineralization. The calcified eggshell has been extensively investigated in the chicken. It is characterized by an inventory of more than 900 matrix proteins. In addition to proteins involved in shell mineralization and regulation of its microstructure, the shell also contains numerous antimicrobial proteins and peptides (AMPPs) including lectin-like proteins, Bacterial Permeability Increasing/Lipopolysaccharide Binding Protein/PLUNC family proteins, defensins, antiproteases, and chelators, which contribute to the innate immune protection of the egg. In parallel, some of these proteins are thought to be crucial determinants of the eggshell texture and its resulting mechanical properties. During the progressive solubilization of the inner mineralized eggshell during embryonic development (to provide calcium to the embryo), some antimicrobials may be released simultaneously to reinforce egg defense and protect the egg from contamination by external pathogens, through a weakened eggshell. This review provides a comprehensive overview of the diversity of avian eggshell AMPPs, their three-dimensional structures and their mechanism of antimicrobial activity. The published chicken eggshell proteome databases are integrated for a comprehensive inventory of its AMPPs. Their biochemical features, potential dual function as antimicrobials and as regulators of eggshell biomineralization, and their phylogenetic evolution will be described and discussed with regard to their three-dimensional structural characteristics. Finally, the repertoire of chicken eggshell AMPPs are compared to orthologs identified in other avian and non-avian eggshells. This approach sheds light on the similarities and differences exhibited by AMPPs, depending on bird species, and leads to a better understanding of their sequential or dual role in biomineralization and innate immunity.

## 1 Introduction

Birds lay eggs in which all internal constituents are protected against the external environment by a mineralized structure, the calcitic eggshell, which protects the developing embryo from physical shocks and microbial contamination (1, 2). Eggshell pigmentation allows thermal regulation of the internal egg components and embryo, while the outer proteinaceous cuticle limits water loss and pathogen ingress through eggshell respiratory pores (3, 4). The avian egg is an impressive terrestrial adaptation to resist the desiccating nonaquatic environment. The chicken eggshell is composed of calcium carbonate (95%), an organic matrix comprising various proteins (3.5%), including glycoproteins and proteoglycans, and water. These proteins are expressed and secreted at specific time points during eggshell calcification (1, 5) (initial, growth and termination phases) and guide formation of the unique eggshell ultrastructure and microstructure (6). During these distinct phases of eggshell calcification, the matrix proteins play a key role to stabilize and transport amorphous calcium carbonate (ACC) transient form of calcium carbonate, which transforms into the calcite polymorph (2). The mechanism by which these proteins interact with the mineral phase is under active investigation and is likely to rely on their three-dimensional structures and/or physicochemical properties (7).

## 2 Eggshell Structure and Formation

The distinctive features of the avian eggshell, as compared to bone or teeth, are the nature of the mineral deposit - calcium carbonate in the form of calcite, as well as the absence of cell-directed assembly during its fabrication (1). The avian eggshell is remarkable for its extreme mechanical properties. All avian eggshells consist of the trigonal phase of calcium carbonate, in the calcite crystalline form, which is its most stable polymorph at room temperature. In most bird species, the mass of eggshell is proportional to the egg mass (8) and represents 10-11% of egg weight. The chicken eggshell has been the most studied to date. It contains 1.6% water, 3.3 to 3.5% organic matrix when the eggshell membranes are included and 95% calcium carbonate. The minor mineral ions possess a heterogeneous distribution: Mg is higher in the mammillary layer and at the outer palisade layer, while phosphorus (as inorganic phosphate or associated with phosphoproteins) is mainly incorporated during the eggshell termination phase and is found in the outer palisade layer and cuticle (2, 7, 9). In addition, the eggshell contains glycosaminoglycan moieties (10, 11) as proteoglycan constituents including hyaluronic acid (48%) and galactosaminoglycans (52%: chondroitin sulfate, dermatan sulfate) that have been demonstrated to influence calcium carbonate crystallization *in vitro* (12–16).

**Figure 1:**
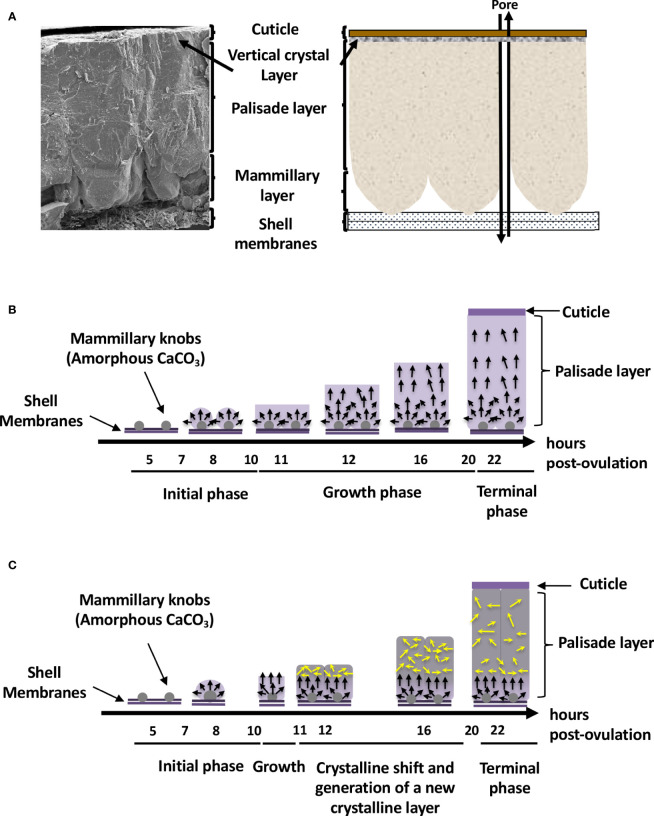
Avian eggshell structure and kinetics of its mineralization events. **(A)** Scanning electron microscope photography (left) and diagram (right) showing the six different layers composing the avian eggshell. From inside to outside are the two proteinaceous eggshell membrane layers supporting the calcified region of the shell (mammillary, palisade and vertical crystal layers). The most external zone is the thin organic cuticle layer. **(B)** Scheme representing shell mineralization pivotal stages that determine the eggshell ultrastructure and crystallographic texture in most bird species. They correspond to the initial stage dominated by amorphous calcium carbonate (ACC) deposition on eggshell membranes, its progressive transformation to form calcite aggregates on mammillary knobs, and the growth of large calcite units. The last stages correspond to the formation of the columnar calcite crystals and the deposition of cuticle. Black arrows describe the orientation of crystals that compete for the available space and only those favorably oriented nearly perpendicular to the shell surface (along the c-axis), are selected and contribute to the development of a preferential orientation of crystals. Timing (hours post-ovulation) are given for the kinetics of chicken eggshell fabrication. **(C)** Specific bilayer structure of the Guinea fowl eggshell. Guinea fowl eggshell mineralization initially follows the same pattern described in chicken resulting in the characteristic columnar structure (black arrows). However, a sharp change in the size and orientation of crystals occurs (yellow arrows) at 11 hours post ovulation, when one-third of the final eggshell thickness has been deposited. At this point, the large columnar calcite units break up into smaller crystal units with varying crystallographic orientations forming a microstructure with an intricate interlacing of calcite crystals, leading to the formation of a secondary layer with misoriented crystals.

From inside to outside, six different eggshell layers can be distinguished (1) (**Figure 1A**). In chicken, the eggshell is about 400 µm thick. The innermost layers are made of two eggshell membranes composed of interlacing protein fibers. The inner shell membrane is 20 µm thick and is in contact with the egg white. The mineralization of the shell is initiated and grows outward from the outer shell membrane (50 µm thick) to form the mammillary layer (70 µm thick), which constitutes the innermost part of the calcified layer (**Figure 1B**). Its base consists of the mammillary knobs, which are organic clusters, deposited on the surface of the outer shell membrane. Mineralization starts from these mammillary knobs and continues outwards, initially forming cone-like structures that fuse to form the columnar palisade layer (**Figure 1B**). Both the mammillary and palisade layers contain occluded organic matter. The mineralized eggshell contains respiratory pores that span its thickness and allow the gas exchange required for embryonic respiration. A surface layer of small adjacent single calcite crystals (vertical crystal layer) is then deposited vertically on the surface of the palisade layer, followed by the proteinaceous cuticle (**Figures 1A, B**). The cuticle enters the funnel-shaped mouth of each respiratory pore, forming a plug, which prevents the penetration of bacteria into the egg and regulates water loss, while allowing exchange of metabolic gases (3).

The eggshell’s main ultrastructural characteristics (columnar shape) and mineralogical composition (calcite) are similar in most bird species. The Guinea fowl is one notable exception. Its eggshell is about 500 µm thick and calcification follows the same pattern as for other birds; however, a novel change in the size and orientation of crystals occurs approximately in the middle of the calcified layer (17, 18) (**Figure 1C**). In the Guinea fowl eggshell, large columnar calcite units break into smaller crystal units with varying crystallographic orientation forming a microstructure with an intricate interlacing of calcite crystals (**Figure 1C**). This particular structure is responsible for the enhanced mechanical properties of Guinea fowl eggshell by comparison to those of other bird species (19, 20).

The forming egg acquires its different layers in specific segments of the oviduct. After ovulation, the yolk enters the infundibulum where the outer perivitelline layer is secreted. The egg white and the shell membranes are next deposited, in the magnum and the white isthmus regions, respectively. Shell mineralization begins when the forming egg enters the uterine (shell gland) portion of the oviduct, five hours after ovulation. During shell mineralization, the egg is bathed in an uterine fluid that contains the necessary organic and mineral precursors (21). The transition of ions to a crystalline state is achieved through amorphous transitional forms allowing crystallisation under physiological conditions. In birds, calcium carbonate is initially deposited as an amorphous calcium carbonate phase (ACC), which progressively converts into calcite (22). Matrix proteins stabilize ACC, promote crystal nucleation, select the calcite polymorph, and regulate the evolution of crystal size and morphology (1, 2, 6). These matrix–mineral interactions determine the orientation of calcite crystals, which results in the complex ultrastructure of the eggshell, its peculiar texture, and consequently, its mechanical properties. Shell formation is temporally controlled, and in chickens, four main steps have been identified during the 17 h process (from 5 h to 22 h post-ovulation) (**Figure 1B**) (22). They correspond to the initial stages that are dominated by ACC deposition on the outer eggshell membrane, its progressive transformation to form calcite aggregates on mammillary knobs surrounded by ACC particles, the growth of large calcite units surrounded by ACC, and the final deposit of cuticle. Calcite crystals rapidly grow to form larger crystal units. The interaction with eggshell organic matrix components inhibits growth of calcite crystal faces parallel to the c-axis, thus causing elongated crystal growth in this preferential direction. Calcite crystals growing with their c-axis nearly perpendicular to the eggshell surface block the growth of adjacent crystals with less favourable orientations, resulting in the development of columnar calcite units. Finally, mineralization is terminated and the thin proteinaceous cuticle is deposited on the shell surface (1, 22).

## 3 Avian Eggshell Antimicrobial Proteins and Peptides

Early investigations of extracted eggshell matrix proteins were focused on their ability to modify crystal growth patterns from super-saturated calcium carbonate solutions *in vitro*. However, the identification of ovotransferrin and lysozyme C as eggshell matrix proteins suggested they could additionally play a role in antibacterial protection (23, 24). Moreover, soluble shell extracts possess antimicrobial activity, and were demonstrated to inhibit the growth of three bacteria species, *Pseudomonas aeruginosa (P. aeruginosa)*, *Bacillus cereus (B. cereus)*, and *Staphylococcus aureus (S. aureus)* (25). However, the antibacterial activity of the eggshell matrix is likely not solely explained by the presence of lysozyme C and ovotransferrin, considering their relative low abundance, and it was predicted that other antibacterial proteins probably act in synergy. Subsequently, antimicrobial activities were characterized for eggshell matrix proteins such as ovocalyxin-36 (OCX-36) (26–28), ovocalyxin-32 (OCX-32) (29, 30) and ovocleidin-17 (OC-17) (31). Integration between the eggshell proteomic and antimicrobial literatures allowed the identification of at least twenty eggshell proteins with antimicrobial activities (**Table 1**). Their antimicrobial activities rely on either direct or indirect mechanisms of action. For example, lectins, BPI/LBP/PLUNC proteins, defensins, muramidases, MSMB3 and pleiotrophin can potentially interact with the bacterial surface components, triggering lipopolysaccharide, peptidoglycan and/or membrane destabilization, followed by bacterial growth inhibition or death (**Table 1**). On the other hand, the antimicrobial activity of other proteins, such as inhibitors of microbial proteases or chelators of iron or biotin, is rather indirect (**Table 1**). Of these 20 proteins, 13 are cationic with a pI > 8.0 (**Table 1**). Moreover, certain proteins (VMO1, AvBD11, OVAX, MSMB3 and pleiotrophin) are able to bind sugar residues (38) (**Table 1**). The highly cationic heparin-binding domain of these proteins seems to be required for antibacterial activity but might also allow interaction with eggshell proteoglycans.

**Table 1:** List of antimicrobial proteins and peptides identified in chicken eggshell by mass spectrometry.

Family/Protein type	Protein name (Alternative name)	Gene-ID / UniprotKB accession	3D structure PDB accession	Molecular weight / pI value	Known ligand(s) or substrate(s)	Antibacterial activity	Other antimicrobial activity	Proteomic identification		
								Eggshell membrane proteome	Calcified layer proteome	Cuticle proteome
**Lectin (C-type lectin)**	Ovocleidin-17 (OC-17)	gene not assigned / Q9PRS8	1GZ2	17 kDa / 11.6	Bacterial polysaccharide	*Bacillus subtilis, Staphylococcus aureus, Pseudomonas aeruginosa*		*	*	*
**Lectin (β-prism lectin)**	Vitelline membrane outer layer protein 1 (VMO1)	418974 / P41366	1VMO	18 kDa / 10.0	GAG (heparin), hexasaccharides of N-acetylglucosamine	*Listeria monocytogenes*		*	*	
**BPI/LBP/PLUNC**	Ovocalyxin-36 (OCX-36)	419289 / Q53HW8	Homology model	46 kDa / 6.0	LPS, LTA	*Staphylococcus aureus*		*	*	*
	TENP	395882 / O42273	Homology model	46 kDa / 6.0		*Micrococcus luteus, Bacillus subtilis*		*	*	*
**Defensin**	AvBD11 (gallinacin-11, VMO-II)	414876 / Q6IV20	6QEU (6QES=Nter domain, 6QET=Cter domain)	9.3 kDa / 10.4 (Nter domain : 12.2 ; Cter domain : 9.0)	GAG (heparin)	*Listeria monocytogenes, Salmonella enterica* Enteritidis	Antiparasitic, antiviral	*	*	
	AvBD10 (gallinacin-10)	414341 / Q6QLQ9	n.d	4.4 kDa / 8.4		*Enterococcus faecalis, E. coli*		*	*	
	AvBD9 (gallinacin-9)	414343 / Q6QLR1	n.d	4.4 kDa / 8.9		*Enterococcus faecalis, E. coli*		*		
	Ovodefensin A1 (gallin, OvoDA1)	422030 / R4GI90	2MJK	5 kDa / 10.4		*E. coli, Staphylococcus aureus*		*		
**Muramidase**	Lysozyme C	396218 / P00698	1SFA	14.3 kDa / 11.0	Peptidoglycan, chitin, Carbohydrates of various length	Gram+ / Gram- bacteria	Antifungal, antiviral	*	*	*
	Lysozyme G	395708 / P27042	154L (goose)	20 kDa / 10.4	Peptidoglycan, chitin, Carbohydrates of various length	Gram+ / Gram- bacteria			*	
**Antiprotease**	Ovoinhibitor	416235 / P10184	n.d	50 kDa / 6.4	Serine proteases	*Bacillus thuringiensis*		*	*	*
	Chicken cystatin (Ovocystatin)	396497 / P01038	1CEW	12 kDa / 7.3	Cysteine proteases	*Porphyromonas gingivalis, E. coli,Pseudomonas aeruginosa, Acinetobacter lowffii, Oligella sp.*	Antiparasitic, antiviral, antifungal	*	*	*
	Ovocalyxin-32 (OCX-32)	395209 / Q90YI1	Homology model	32 kDa / 9.0	Carboxypeptidases	*Bacillus subtilis*		*	*	*
	Ovalbumin-related Protein-X (OVAX)	420898 / R9TNA6	7QRN (on hold)	45 kDa / 8,5	GAG (heparin)	*Listeria monocytogenes,Salmonella enterica* Enteritidis		*	*	*
	Ovostatin (Ovomacroglobulin)	396151 / P20740	n.d	162 kDa / 5.6		*Serratia marcescens, Pseudomonas aeruginosa*		*	*	
**Chelator**	Avidin	396260 / P02701	2AVI	17 kDa (monomer) / 10.0	Biotin (vitamin H, vitamin B8)	Biotin-requiring bacteria		*	*	
	Ovotransferrin (Conalbumin)	396241 / P02789	1N04	78 kDa / 7.3	Iron (Fe^3+^), carbonate ion	*S. aureus, B. cereus*, Gram- bacteria	Antiviral (Marek disease virus)	*	*	*
	Extracellular fatty acid binding protein (Ex-FABP, lipocalin 8)	396393 / P21760	3SAO	18 kDa / 6.3	Iron (Fe^3+^) sequestered in siderophores, LPA	*E. coli*, *B. subtilis*		*	*	
**Other**	β-microseminoprotein-like 3 (MSMB3)	101750704/ n.a	6RWC	10 kDa (monomer) / 10.4	GAG (heparin)	*Listeria monocytogenes, Salmonella enterica* Enteritidis		*		
	Pleiotrophin	418125 / P32760	Homology model	15 kDa (monomer) / 11.0	GAG (heparin), chondroitin sulfate A, syndecan	*Listeria monocytogenes, Salmonella enterica* Enteritidis			*	

Isoelectric points (pI) values have been calculated using the Poisson-Boltzmann method implemented in PDB2PQR software (32) (https://server.poissonboltzmann.org/pdb2pqr) with the 3D coordinate file of each protein. pK_a_ values were calculated using PROPKA protonation states assignment at pH 7.0.

For TENP, AvBD10, AvBD9, ovoinhibitor and ovostatin, pI values were calculated from the protein sequence using Protparam tool at expasy.org.

GAG, LPS, LTA, LPA, stand for glycosaminoglycan, lipopolysaccharide, lipoteichoic acid and lysophosphatidic acid, respectively.

n.d: the 3D structure has not been determined and comparative modeling would be of low confidence.

AMPPs were identified from published proteomes of eggshell membrane (33, 34), calcified layer (35) and cuticle (36, 37).

The cationic features of several eggshell antimicrobial proteins and their distribution in many eggshell layers (eggshell membranes, calcified eggshell and the cuticle, **Table 1**) suggest that these proteins might also have a function in biomineralization, by interacting with eggshell proteoglycans and with ions of the mineral phase. Such dual activities have been already described for three of these antimicrobial proteins, namely ovotransferrin (an iron-chelator), OC-17 (a C-type lectin), and lysozyme C (an enzyme with muramidase activity) (**Table 1**). Indeed, in addition to their respective antibacterial activity, there is evidence that eggshell matrix components can influence the morphology and size of calcite crystals grown *in vitro* (20, 21, 30, 31) or stabilize ACC (39, 40).

### 3.1 Lectin-Like Proteins

#### 3.1.1 Ovocleidin-17 (OC-17)

The C-type lectin ovocleidin-17 (OC-17) is an eggshell-specific protein, which was first purified and partially sequenced from the chicken eggshell (41). Its GC-rich mRNA sequence was determined only recently by *de novo* transcriptomic assembly (42). OC-17 contains a C-type lectin (CTL) domain and possesses two phosphorylated serine residues. CTL proteins are a huge family of proteins including at least seven subgroups such as hyalectans, asialoglycoprotein receptors, collectins, selectins, natural killer group transmembrane receptors, macrophage mannose receptors and simple lectins (43). OC-17 and its orthologs in other birds correspond to a simple lectin, with a short amino acid sequence (about 150 amino acids) and only one CTL domain. OC-17 orthologs have been identified in the eggshell of many bird species, including ostrich, emu, and rhea (44, 45). In each of these ratites, two homologous CTL eggshell proteins were identified and named according to the bird species: struthiocalcin-1 and 2 (SCA-1 and -2) for ostrich (*Struthio camelus*), dromaiocalcin-1 and -2 (DCA-1 and -2) for emu (*Dromaius novaehollandiae*) and rheacalcin-1 and -2 (RCA-1 and -2) for rhea (*Rhea americana*). Recently, the complete gene structure of two paralogous OC-17-like genes were reported for duck (anascalcin-1, ACA-1; anascalcin-2, ACA-2) (46). Corresponding protein names have recently been given to their paralogs found in various bird species: XCA-1 and XCA-2 (47). Moreover, *de novo* assembled transcriptomic data from multiple tissues in five bird species (chicken, duck, pigeon, zebra finch, and goose) revealed newly identified OC-17-like cDNAs in these species, suggesting that the duplicated OC-17-like family members have been conserved during avian speciation (46). For example, two CTL proteins are identified in the Guinea fowl eggshell proteome (annotated as OC-17-like and DCA-1-like) (19). However, only one eggshell-specific CTL protein (OC-17) has yet been identified in chicken. Proteomics analysis demonstrated that OC-17 is a highly abundant protein in the eggshell matrix in chicken and Guinea fowl (OC-17-like) (19, 20).

**Figure 2:**
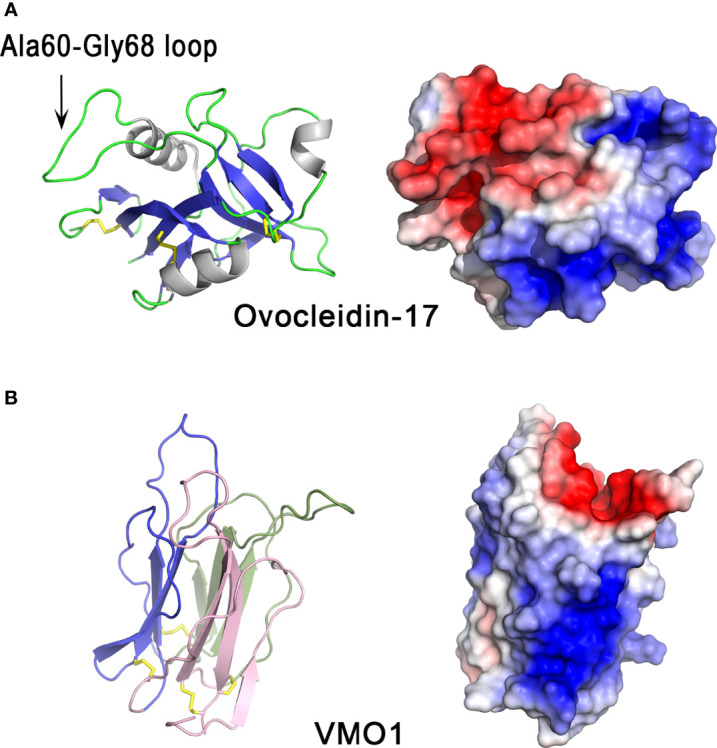
3D structures of eggshell lectin-like proteins, ovocleidin-17 and VMO1. 3D structure of chicken ovocleidin-17 **(A)** and chicken VMO1 **(B).** The left panels correspond to the cartoon representation of 3D structure while the right panels show the color-coded electrostatic potential molecular surface. Color scheme ranges from red (negatively charged regions) to deep blue (positively charged regions). The Ala60-Gly68 exposed loop of ovocleidin-17 containing the two phosphorylatable serine residues (Ser61 and Ser67) is indicated. The figure was prepared using Pymol software (48) and APBS (Adaptive Poisson-Boltzmann Solver) plugin (49) for electrostatic calculations using atomic coordinates of 1GZ2 and 1VMO PDB files (www.rcsb.org) for OC-17 and VMO1, respectively.

The cartoon representation of ovocleidin-17 (OC-17) structure (**Figure 2A**) shows that the molecule has a mixed α/β structure containing a single C-type lectin-like domain which is composed of three α-helices and two antiparallel β-sheets, each made up of four strands. The Ala60-Gly68 loop exposed region contains the two phospho-accepting serine residues (Ser61 and Ser67). The crystal structure of OC-17 indicate that charge distribution on the molecular surface of OC-17 is rather asymmetrical (**Figure 2A**). On one side of the molecule, the surface of the protein displays an extended solvent-exposed basic hotspot consisting of 17 of the 21 arginine/lysine residues of the molecule. On the opposite side, the two phosphate groups associated with phospho-Ser61 and phospho-Ser67 bring additional negative charges together with Asp and Glu residues in or in the vicinity of the Ala60-Gly68 solvent-exposed loop region. This suggests that these OC-17 positively and negatively charged clusters could interact with either carbonate ions or calcium ions of the mineral phase during the biomineralization process. *In silico* molecular dynamics simulations suggest that OC-17 may adopt at least three different conformations, one of these being able to bind calcium carbonate surfaces through its positively charged guanidino group of specific arginine residues (50, 51). Indeed, purified OC-17 was shown to modify calcite crystallization *in vitro* (52). Thus, CTL proteins, which are highly conserved in bird eggshell, could play a role in eggshell formation by binding to specific calcite crystal faces (53). Moreover, CTL proteins have also been identified in the biomineralization process of invertebrates. For instance, in the sea urchin *Strongylocentrotus purpuratus*, SM50 is a protein containing a CTL domain, in addition to glycine-rich and proline-rich regions, and has been demonstrated to influence CaCO_3_ biomineralization (54). Likewise, in the freshwater pearl mussel, the CTL protein perlucin, also identified in the shell proteomes of other mollusks, is involved in nacre formation (55). In birds and reptiles, OC-17-like paralogous genes are clustered together in syntenic genomic regions; however, they are absent from the same locus in mammalian and amphibian genomes, supporting the hypothesis that OC-17-like paralogous genes encoding eggshell-specific proteins are specific to reptiles and birds that produce a calcified eggshell (47).

In addition to its role in biomineralization, chicken OC-17 exhibits antimicrobial activity, and thus could play a role in innate immunity (31). Purified ovocleidin-17 binds bacterial polysaccharides, and is bactericidal against *B. subtilis*, *S. aureus* and *P. aeruginosa* (31). Antimicrobial activity was found to be enhanced in the presence of calcium. The highly positively charged region of OC-17 is likely to be involved in the interaction of OC-17 with the bacterial cell wall, with their subsequent disruption, as observed for many cationic antimicrobial peptides (56).

#### 3.1.2 Vitelline Membrane Outer Layer Protein 1

VMO1 is a cationic protein of about 18 kDa, initially identified in the outer layer of the egg perivitelline layer (vitelline membrane) of the chicken egg yolk, where it is one of the major components, in addition to ovomucin, lysozyme and AvBD11 (57). However, VMO1 is also present in egg albumen (58) and in eggshell (35). This protein is synthesized in the oviduct, particularly at the infundibulum-magnum junction (59) responsible for the formation of perivitelline layer and/or the albumen, and its synthesis can be induced by estrogen (60), like most major egg white proteins. Its abundance in the eggshell, however, is low (35).

Structurally, the VMO1 fold is characterized by three β-sheets arranged like the three faces of a prism (**Figure 2B**, left panel). This “β-prism” fold has three β-sheets with Greek key motifs arranged around a three-fold symmetry axis (61), which perfectly reflect the occurrence of three internal repeats of about 53 residues in the amino acid sequence. The whole arrangement is stabilized by internal hydrophobic interactions arising from inter β-sheet contacts and by four disulfide bonds at the base of the β-prism. The solvent-accessible surface (**Figure 2B**, right panel) shows a strongly biased charge distribution with a positive cluster, probably involved in the interaction with negatively charged glycosaminoglycans and/or negatively charged bacterial membranes. The negative cavity at the top of the prism appears to be suitable for binding of simple sugars or oligosaccharides as observed in the corresponding cavity in mannose-lectin or galactose-lectin complex of many lectins with β-prism fold. This charge distribution strikingly resembles that of OC-17. The β-prism fold is found in other proteins, in particular plant lectins related to jacalin (known to specifically bind mannose or galactose) (62) and domain II of the δ-endotoxin (insecticidal protein of *Bacillus thuringiensis*), which interacts with N-acetylgalactosamine (63).

VMO1 possesses carbohydrate-binding properties, in addition to a transferase-like enzymatic activity that catalyzes glycan synthesis (synthesis of N-acetylchito-oligosaccharides from an N-acetylglucosamine hexasaccharide), and inhibits hemagglutination induced by the lectin wheat germ agglutinin, presumably by competing for binding to the same sugars as those recognized by the lectin (64). VMO1 also binds to heparin (negatively charged glycosaminoglycan) and has moderate antibacterial activity against *Listeria monocytogenes*, which might partly involve the heparin-binding site(s) of the protein (38). Despite this observation, the main physiological function of VMO1 remains uncertain. In chicken, VMO1 has been detected not only in the egg but also in rooster seminal plasma and spermatozoa (65), suggesting that it has other roles in avian reproduction (fertilization).

VMO1 homologs were also identified in mammals, in particular in camel lacrimal fluid (66), where it can associate with lysozyme and stabilize the tear film (67). In addition, transcripts encoding VMO1 have been found in a specific region of the sheep hypothalamus, which exhibit photoperiod-depend regulation (68), and also in the Lichtenstein’s green racer (*Philodryas olfersii)* snake venom gland (69). These latest observations suggest that the physiological functions of VMO1 may differ depending on the species and/or the tissue specificity.

### 3.2 BPI/LBP/PLUNC Family Proteins: Ovocalyxin-36 and TENP

Ovocalyxin-36 (OCX-36) is a prominent 36 kDa protein present in the uterine fluid collected during the active calcification stage of chicken eggshell mineralization (27). OCX-36 mRNA is expressed in the chicken oviduct segments where eggshell formation takes place (isthmus and uterus), and its expression is strongly upregulated during eggshell calcification. OCX-36 was initially thought to be eggshell-specific; however, in addition to the distal oviduct, it is also expressed in the chicken intestine (70).

The OCX-36 protein sequence is composed of two lipid-binding domains BPI1 (BPI/LBP/CETP N-terminal domain) and BPI2 (BPI/LBP/CETP C-terminal domain) of about 200 amino acids each (27, 47, 71). This protein displays significant identity with mammalian lipopolysaccharide-binding proteins (LBP), bactericidal permeability-increasing proteins (BPI) and palate, lung and nasal epithelium clone (PLUNC) family proteins that are key components of the innate immune system and act as a first line of host defense (27, 71). LBP proteins initiate the inflammatory host response upon the detection of a pathogen. OCX-36 may therefore participate in natural defense mechanisms that keep the egg and oviduct free of pathogens. This hypothesis is supported by observations that purified OCX-36 protein binds bacterial lipopolysaccharide (LPS) and lipoteichoic acid (LTA), and inhibits *S. aureus* bacterial growth (26). Purified OCX-36 and OCX-36-derived peptides differentially modulate innate immune responses *in vitro* (macrophage cell culture) and *in vivo* (mouse model of endotoxemia) (28).

**Figure 3:**
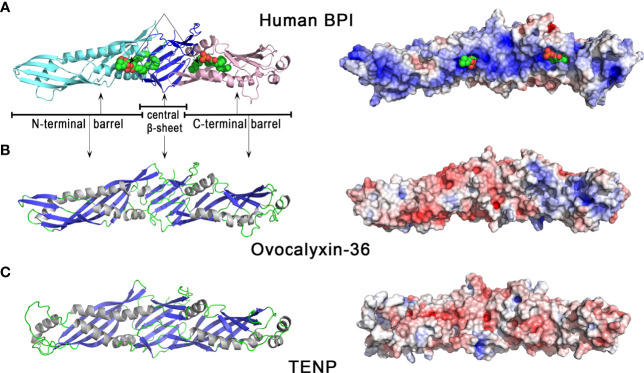
3D structures of human BPI and chicken eggshell BPI/LBP/PLUNC proteins, ovocalyxin-36 and TENP. 3D structure of human BPI **(A)**, chicken ovocalyxin-36 **(B)**, and chicken TENP **(C)**. The left panels correspond to the cartoon representation of 3D structure while the right panels show the color-coded electrostatic potential molecular surface. Color scheme ranges from red (negatively charged regions) to deep blue (positively charged regions). The figure was prepared using Pymol software (48) and APBS (Adaptive Poisson-Boltzmann Solver) plugin (49) for electrostatic calculations using atomic coordinates of 1BP1 PDB file (www.rcsb.org) for human BPI. The structure of ovocalyxin-36 was predicted by homology modeling using SwissModel server (swissmodel.expasy.org) and human BPI as a template. The 3D structure of TENP was modeled using the AlphaFold algorithm and was retrieved in the AlphaFold protein structure database under accession number AF-O42273-F1 (72, 73).

The three-dimensional structure of OCX-36 has not yet been determined; however, a 3D homology model based on the published 3D structure of human BPI (**Figure 3**) is available. Human BPI (**Figure 3A**) is a boomerang-shaped molecule consisting of two domains of similar size connected by a proline-rich linker region giving the protein a pseudo-twofold symmetry. Structurally, these two domains contain a barrel at each N-terminal and C-terminal end. Both barrels have a similar topology despite dissimilar sequences (< 20% sequence identity). A central β-sheet interacts with both barrels. Interestingly, two phosphatidylcholine molecules are bound to BPI, each of them in a hydrophobic cavity on the concave surface of BPI (**Figure 3A**). Since phosphatidylcholine share some structural similarity with LPS, it has been suggested that acyl chains of LPS could be able to bind in these apolar pockets (74). The 3D homology model of chicken OCX-36 (**Figure 3B**) reveals that the corresponding phospholipid binding sites are bordered by hydrophobic residues and thus may bind phospholipids or acyl chains of LPS.

The “transiently expressed in neural precursors” (TENP) gene product is another member of the bacterial/permeability-increasing (BPI) family of antimicrobial proteins which was first identified in post-mitotic cells during early neurogenesis (75). In chicken oviduct, the expression of TENP is largely confined to the tubular glands of the magnum, where egg white synthesis occurs (76). TENP protein was identified in chicken egg white (58, 77, 78), and can be purified by affinity chromatography with other egg white heparin-binding proteins, a property which is a hallmark of some antimicrobial proteins (38). In emu, the TENP gene is highly expressed in the magnum of the oviduct, and the protein is a major egg white component. Purified emu TENP exhibits antibacterial activity against Gram-positive bacteria, including *Micrococcus luteus* (*M. luteus*) and *B. subtilis*, but not against Gram-negative bacteria such as *E. coli* and *Salmonella* Typhimurium (79). Similar to chicken ovocalyxin-36, TENP retains the two-domain structure of BPI, despite quite low sequence identities with BPI (about 20%). Like OCX-36, the phospholipid binding sites seen in human BPI (**Figure 3A**) are also present in TENP (**Figure 3C**), which suggests that they may bind phospholipids or acyl chains of LPS. However, chicken TENP is less cationic than its human counterpart, as shown by the distribution of electrostatic potential values at the surface of the two molecules (**Figures 3C** versus **3A**, right panels). TENP and OCX-36 have the same theoretical pI value (pI = 6.0, **Table 1**) but the distribution of electrostatic charges is slightly different, with a cationic area in the C-terminal domain seen in OCX-36 structure that is lacking in TENP (**Figure 3B** vs **3C**, right panels).

The BPI/LBP/PLUNC protein family belongs to the TULIP (tubular lipid-binding) superfamily, which split into two groups before the last eukaryote common ancestor: SMP-like proteins (synaptotagmin-like, mitochondrial and lipid-binding proteins) and BPI-like proteins (80). In vertebrates, the similar organization of exons/introns in members of the BPI/LBP/PLUNC family, as well as synteny analysis, strongly suggests a common origin for the genes encoding chicken OCX-36, TENP and other BPI family B members. All these genes might have arisen by multiple duplication events (81). Phylogenetic analysis reveals the presence of an OCX-36 orthologous gene in reptiles (turtle and alligator), and other bird species, including, for example, Palaeognathae (kiwi), Neoaves (zebra finch), and Galloanserae (duck). In addition, analysis of the platypus genome (*Ornithorhynchus anatinus*), an egg-laying mammal (Monotremata), reveals the presence of BPIFB4-like gene at the same gene locus as OCX-36 in birds and reptiles (47). Therefore, OCX-36 appeared before the divergence of birds and mammals, and was likely lost in therian mammals (placentals and marsupials). Moreover, it is likely that TENP is the oldest gene in the BPI/LBP/PLUNC family, and that the OCX-36 gene is the result of three duplication events before tetrapod diversification and one event in amniotes (47). Thus, the potential role of OCX-36 and TENP is associated with the innate defense of the egg against pathogens, and egg-laying animals diversified their antimicrobial role in egg protection through a duplication-driven adaptive process (81).

### 3.3 Defensins : AvBD11, AvBD9, AvBD10, OvoDA1

Defensins are cysteine-rich cationic antimicrobial peptides found in a wide range of living organisms (vertebrates, invertebrates, plants, fungi) (82). Avian defensins belong to the β family of vertebrate defensins and are grouped in two different gene clusters, namely the avian β-defensins (AvBDs) and the ovodefensins (OvoDs), both being located on chromosome 3 in the chicken genome.

Several defensins have been identified in chicken eggshell proteomes, including AvBD9, AvBD10, AvBD11 and OvoDA1 (**Table 1**). AvBD11 and AvBD10 are present in both the calcified layer and eggshell membranes, while AvBD9 and OvoDA1 have been reported only in eggshell membranes. All these defensins are synthesized in the oviductal segment responsible for eggshell calcification (79, 80). An *in vitro* study revealed that AvBD9, AvBD10 and AvBD11 are among the AvBDs constitutively expressed in chicken oviduct epithelial cells isolated from the isthmus of laying hens (83). The oviductal expression of some egg defensins might be responsive to microbial stimulation. In this regards, AvBD10 gene was shown to be up- or down-regulated by Toll-Like Receptor ligands in cultured mucosal tissues from the uterus (84). It is noteworthy that AvBD11 and OvoDA1 are more abundant in egg white and perivitelline layer (vitelline membrane) than in eggshell. Accordingly, AvBD11 and OvoDA1 mRNA are predominantly detected in magnum (oviductal segment responsible for the synthesis of egg white proteins), but also, to a lesser extent, in the isthmus (85, 86). The oviductal expression of AvBD11 and OvoDA1 genes is regulated by sex hormones such as estrogen and progesterone (86, 87), similar to most abundant egg white proteins. All four of these defensins possess a cationic pI (**Table 1**).

**Figure 4:**
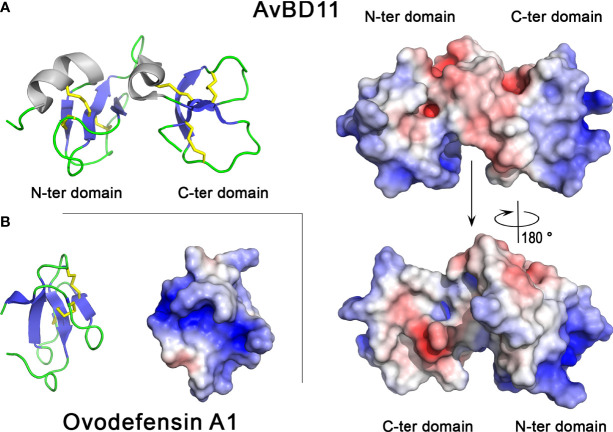
3D structures of chicken defensins AvBD11 and OvoDA1. 3D structure of chicken AvBD11 **(A)** and chicken OvoDA1 **(B).** The left panels correspond to the cartoon representation of 3D structure while the right panels show the color-coded electrostatic potential molecular surface. Color scheme ranges from red (negatively charged regions) to deep blue (positively charged regions). The figure was prepared using Pymol software (48) and APBS (Adaptive Poisson-Boltzmann Solver) plugin (49) for electrostatic calculations using atomic coordinates of 6QEU and 2MJK PDB files (www.rcsb.org) for AvBD11 and OvoDA1 structure, respectively.

At the structural level, β-defensins adopt a three-stranded antiparallel β-sheet fold stabilized by three disulfide bonds that are arranged according to the typical disulfide array C1-C5/C2-C4/C3-C6 of β-defensins. Although their 3D structure has not yet been solved, AvBD9 and AvBD10 are predicted to adopt the fold and disulfide-bridge topology of β-defensins. AvBD11 (82 amino acids, 9.3 kDa for the mature form) is unique among bird defensins as it consists of two β-defensin domains (**Figure 4A**, left panel) (88, 89). Chicken AvBD11 is the archetype of the structural avian double β-defensin family and such a double-β-defensin has never been identified in mammals, yet. Both N- and C-terminal moieties form independent domains adopting the typical β-defensin fold, i.e. a three-stranded antiparallel β-sheet stabilized by three disulfide bridges (**Figure 4A**). Hydrophobic interactions between both domains help to maintain a compact structure. The ovodefensin OvoDA1 (formerly named gallin) has a single β-defensin domain. It contains a short two-stranded β-sheet in addition to the three-stranded antiparallel β-sheet and the disulfide bridge array typical of β-defensins (**Figure 4B**). This observation suggests that this egg defensin and presumably the other ovodefensins, form a new structural subfamily of β-defensins (90).

Both AvBD11 and OvoDA1 defensins are highly cationic (pI>10.0, **Table 1**). The AvBD11 surface exhibits large positively charged areas while the hinge region is more acidic, while OvoDA1 is characterized by a large cationic groove (**Figure 4B**, right panel). The cationic properties of defensins and, more generally, antimicrobial peptides, are thought to promote their interaction with the negatively charged bacterial membranes. However, the C-terminal domain of AvBD11 is much less basic (pI = 9.0) than the N-terminal domain (pI = 12.2). This may support that the fact that the N-terminal domain exhibits elevated antibacterial activity compared to the C-terminal domain (88).

All AvBDs, including those reported in the chicken eggshell proteome (AvBD9, AvBD10, AvBD11), possess antibacterial activities (91). The synthetic chicken AvBD9 exhibits antimicrobial activities against various bacteria and yeasts (*Candida albicans* and *Saccharomyces cerevisiae*) (92). Recombinant chicken AvBD9 and AvBD10 display bactericidal activities against *E. coli* (Gram-negative) and *Enterococcus faecalis* (Gram-positive), and the tryptophan residue located at the C-terminal end of AvBD9 contributes to its bactericidal potency (93). Purified chicken AvBD11 binds heparin, and possesses antimicrobial activity against various microorganisms including bacteria (Gram-positive and Gram-negative), *Eimeria tenella* sporozoites and H1N1 influenza virus (38, 88, 89). This double β-defensin also has inhibitory effects on cell growth and/or cell migration (88). Interestingly, the antibacterial, antiparasitic and anti-invasive properties of chicken AvBD11 are mainly associated with the N-terminal β-defensin domain, while the antiviral and cytotoxic activities require the intact native molecule. However, the biological functions of the C-terminal domain still remain unclear, to date. Like AvBDs, OvoDs are also antibacterial (87). In particular, chicken OvoDA1 is active against both *E. coli* and *S. aureus* (Gram-negative and Gram-positive, respectively) (85, 87, 90).

Besides their antimicrobial function, the potential role of these eggshell defensins in the biomineralization process remains unknown. However, it is noteworthy that pelovaterin, a β-defensin-like peptide from the Chinese soft-shelled turtle (*Pelodiscus sinensis*) eggshell matrix, may play a dual role in both antimicrobial egg protection and eggshell calcium carbonate polymorph selection (94). Further studies with chicken eggshell defensins are needed to determine whether they play a role in avian biomineralization.

The AvBD cluster is located in a syntenic region that is conserved in vertebrates, except fish, which suggests that AvBD9, AvBD10 or AvBD11 may have (co)orthologs in a wide range of vertebrate species, including mammals. However, defensin genes have evolved so quickly that it is difficult to precisely define the corresponding orthologs between birds and mammals – currently, only synteny and structural protein similarity has suggested an ancestral link between both groups. In addition, it has to be noted that, to date, no double-β-defensin has been found in mammals. A recent work suggested that AvBD11 probably appeared following a fusion of two ancestral genes or from an ancestral double defensin present before the divergence of Archelosauria, but not from a recent internal duplication (95). On the other hand, OvoD members seem to be restricted to birds and reptiles (87). This particular group of β-defensins is composed of six subfamilies (A to F) based on intra-cysteine amino acid spacing. The A subfamily members (including OvoDA1) have only been detected in birds to date (87). Interestingly, OvoDA1 possesses at least two chicken paralogs (abbreviated OvoDA1-2 and OvoDA3 in the NCBI database) resulting from a relatively recent duplication (85). In chicken, OvoDs are oviduct-specific and assumed to be involved in the antimicrobial protection of the egg (87).

### 3.4 Lysozymes : Lysozyme C, Lysozyme G

#### 3.4.1 C-Type Lysozyme

C-type (chicken-type) lysozyme, or lysozyme C, is a small cationic protein of 14.3 kDa with an isoelectric point of 11.0 (**Table 1**). Although it is highly abundant in chicken egg white, comprising 3.5% of total egg white protein (78), lysozyme C is also present, but at low levels, in eggshell membranes and the calcified layer (24, 35). The chicken lysozyme C gene (abbreviated LYZ) is located on chromosome 1. In the oviduct, it is mainly expressed in the egg white-forming segment (magnum); however, gene expression is also detected, at much lower level, in the red isthmus and in the uterus (24). As for the major egg white proteins, the oviductal expression of the LYZ gene is controlled by steroid hormones (96). Lysozyme C is an enzyme (E.C.3.2.1.17) catalyzing the hydrolysis of the β-(1,4)-glycosidic bond between the N-acetylmuramic acid (NAM) and N-acetylglucosamine (NAG) residues of peptidoglycan, a bacterial cell wall polymer composed of sugars and peptide. This enzymatic activity (muramidase) is the basis for the well-known and potent bacteriolytic effect of lysozyme. Lysozyme C is found in a wide range of animal species, including the phyla Chordata and Arthropoda (97).

Purified egg white lysozyme stabilizes ACC (98) and modifies the morphology of calcite crystals grown *in vitro* (24), which may be physiologically relevant regarding its eggshell localization (eggshell membrane, calcified layer). This observation suggests that lysozyme C could participate in eggshell biomineralization during egg formation, in addition to its role in antibacterial defense. However, eggshell biomineralization occurs in the presence of a complex mixture of matrix proteins, and the precise role of lysozyme still remains uncertain (39, 40).

**Figure 5:**
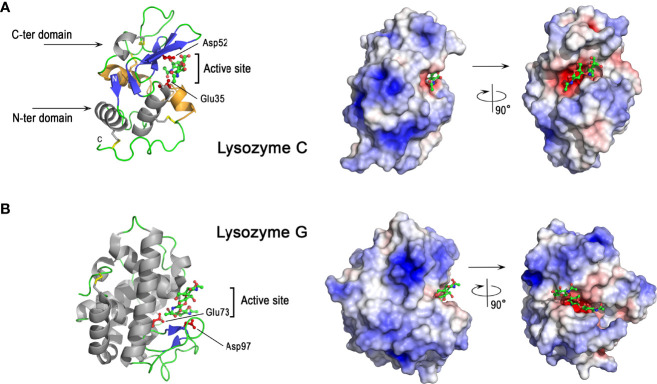
3D structures of avian lysozymes, lysozyme C and lysozyme G 3D structure of chicken lysozyme C **(A)** and goose lysozyme G **(B).** The left panels correspond to the cartoon representation of 3D structure while the right panels show the color-coded electrostatic potential molecular surface. Color scheme ranges from red (negatively charged regions) to deep blue (positively charged regions). The figure shows a disaccharide of N-acetyl-glucosamine bound in the active site of lysozyme C while the structure of lysozyme G is shown as a complex with a trisaccharide of N-acetyl glucosamine (green and red, balls-and-stick representation). The figure was prepared using Pymol software (48) and APBS (Adaptive Poisson-Boltzmann Solver) plugin (49) for electrostatic calculations using atomic coordinates of 1SF4 and 154L PDB file for hen egg white lysozyme C and goose lysozyme G, respectively.

As shown in **Figure 5A**, chicken lysozyme C consists of mixed α and β secondary structures. The molecule has two domains, the N-terminal domain being mainly helical while the C-terminal domain has a three-stranded β-sheet in addition to α-helices. These two domains are separated by a deep cleft containing the catalytic active site of the enzyme. In this site, Glu35 (proton donor) and Asp52 (nucleophile) residues play critical roles in the catalytic mechanism. The exact mechanism, unveiled in 2001 (99), after several decades of debate, implies the formation of a covalently-linked glycosyl-enzyme intermediate during the reaction. Of note, the overall structure of lysozyme is stabilized by four disulfide bonds (**Figure 5A**), which are important for its structure and enzyme activity (100). The peptide corresponding to the Ile98-Arg112 sequence, obtained by clostripain digestion of hen egg white lysozyme, has been shown to retain a broad antimicrobial activity, independent of the enzymatic muramidase activity of lysozyme (101). This peptide corresponds to most of the so-called helix-loop-helix domain (HLH) from Asp87 to Arg114 (colored orange in **Figure 5A** left panel) forming one lip of the active site cleft that binds the sugar ligands, and is highly conserved in vertebrate lysozymes. The lysozyme C surface is highly cationic (**Figure 5A**, right panel), except in the active site region where a hotspot of negative charges in the immediate vicinity of catalytic acidic residues is observed. The deep cleft around the active site allows lysozyme C to bind many oligosaccharides of various length, such as N-acetylglucosamine oligosaccharides (NAGn, n=2 to 6) (**Figure 5A**, right panel).

Lysozyme C possesses antibacterial properties against Gram-positive and Gram-negative bacteria *via* different mechanisms, which are either dependent or independent of its enzymatic activity. Thus the enzyme activity of lysozyme C is not solely responsible for its antibacterial properties. The muramidase activity, responsible for peptidoglycan hydrolysis, is more efficient against Gram-positive bacteria, which possess a thick outer cell wall of peptidoglycan, than against Gram-negative bacteria, for which the peptidoglycan is usually thinner and protected by an outer lipopolysaccharide membrane. Some bacteria are able to develop resistance mechanisms to lysozyme either by modifying their peptidoglycan (N-deacetylation, O-acetylation) (102, 103), or by producing lysozyme inhibitors (104–107). Site-directed mutagenesis demonstrates that lysozyme mutants lacking muramidase activity (Asp52Ser) still retain substantial bactericidal activity against *S. aureus* and *B. subtilis* (108). Lysozyme C can indeed mediate antibacterial effects *via* alternative mechanisms involving the induction of bacterial peptidoglycan hydrolases (autolysins) (109) and/or membrane perturbation effects (108). Lysozyme C also exhibits antibacterial activity against Gram-negative species (110), and can interact with the bacterial cell wall to increase the permeability of the outer and inner membranes, as shown in *E. coli* (111). The antibacterial effect against *E. coli* and other Gram-negative bacteria likely involves the cationic features of the molecule that destabilize the bacterial wall and plasma membrane, as demonstrated for many cationic antimicrobial peptides.

#### 3.4.2 G-Type Lysozyme

Goose-type lysozyme (or lysozyme G) is a cationic protein with a molecular weight of 20 kDa and a pI = 10.4 (**Table 1**). This protein was first isolated from goose egg white (112, 113), and was initially believed to be absent from chicken egg white. Lysozyme G has, however, been detected in the chicken eggshell, but at much lower amounts than lysozyme C (35). The coding gene (LYG2) is located on the chicken chromosome 1, as for the LYZ gene, but in a different locus together and in proximity to two other lysozyme G-like paralogous genes. Lysozyme G is also a muramidase (E.C.3.2.1.17), but its amino acid sequence drastically differs from that of lysozyme C (114). To hydrolyze the β-(1,4)-glycosidic bond between N-acetyl muramic acid (NAM) and N-acetyl-D-glucosamine (NAG) residues of peptidoglycan, lysozyme G uses a different mechanism from that of lysozyme C. The catalytic reaction of lysozyme G involves an inversion of configuration of the chirality of the product, while the configuration of the anomeric center is retained with lysozyme C (99, 115). Some enzymatic specificities have been reported for lysozyme G compared to C-type: it has a distinct preference for NAM residues substituted with a peptide moiety (116), it poorly hydrolyzes the NAG polymer chitopentaose (117) and it is devoid of transglycosylation activity (118). Lysozyme G is detected in many animal species within the phyla Chordata and Mollusca (97).

As shown in **Figure 5B**, lysozyme G shares some structural similarities with lysozyme C despite their amino acid sequence differences. Goose lysozyme G is characterized by the same mixed α and β fold as observed for C-type lysozyme, with a higher proportion of α-helices (**Figure 5B**, left panel). Glu73 of lysozyme G represents the spatial analog of catalytic Glu35 in lysozyme C (119). Lysozyme G lacks an apparent analog to catalytic Asp52 of hen lysozyme C but Asp97 of lysozyme G at the entrance to the active site has been demonstrated to play a key role in catalysis being involved in the inverting-type reaction mechanism (118) together with Glu73. Lysozyme G only contains two disulfide bonds (**Figure 5**). These two bridges are crucial for the structural stability, but not for the correct folding into the enzymatically active conformation, as demonstrated in ostrich lysozyme G (120). The charge distribution onto the molecular surface of lysozyme G is similar to that of lysozyme C (**Figures 5A** vs **5B**, right panels) and reveals the highly cationic properties of both molecules.

### 3.5 Antiproteases : Ovoinhibitor, Cystatin, Ovocalyxin-32, OVAX, Ovostatin

Many protease inhibitors (or antiproteases) are efficient antimicrobials against pathogens, and thus are associated with host defense (121). Their mechanism of action is rather indirect and partly depends on the capacity of their reactive inhibitory site to trap microbial proteases and to block their deleterious activity. Indeed, many of these microbial proteases are major virulent factors that are essential for the survival of viruses (including SARS-CoV-2 virus), bacteria, yeast and parasites, and their dissemination in host organisms. Microbial proteases are often associated with increased pathogenicity, and there is increasing evidence that some pathogen-derived proteases and host protease inhibitors have co-evolved (122).

Similar to proteases, protease inhibitors are assigned to families and clans, based on amino acid similarities and their three-dimensional structure, respectively (123). Protease inhibitors inhibit enzymes using a mechanism that is specific to each family. Five protease inhibitors with reported antimicrobial activity have been identified in the chicken eggshell (20, 35, 124) (**Table 1**): ovoinhibitor, chicken cystatin, ovocalyxin-32 (OCX-32), ovalbumin-related protein X (OVAX) and ovostatin.

#### 3.5.1 Ovoinhibitor

Ovoinhibitor belongs to a family of serine protease inhibitors that encompasses inhibitors possessing one or several Kazal-like units. Their reactive inhibitory site type is extremely variable but the Kazal-type domain is structurally conserved across species: it contains six cysteine residues engaged in disulfide bonds according to a specific pattern (125). Kazal-type inhibitors are widely distributed in all kingdoms of life. Like most small-sized serine protease inhibitors, Kazal-like inhibitors interact with their cognate protease through the so-called “standard mechanism” (126), in which the inhibitory reactive site of the inhibitor specifically interacts with the active site of the cognate protease. This high-affinity interaction leads to the formation of a tight complex and may be accompanied by a slow, but reversible hydrolysis of the inhibitory reactive site loop. Ovoinhibitor contains seven Kazal domains that can inhibit chymotrypsin and trypsin-like proteases (127). Purified inhibitor from egg white exhibits antimicrobial activities against *B. thuringiensis* (127). The relative abundance of ovoinhibitor increases at the terminal stage of eggshell formation (5). Its presence in the cuticle layer (36) suggests that it could partly be released in a soluble form when the outer surface is hydrated, and would act to limit colonization by protease-secreting pathogens. There is no 3D structure available for this inhibitor to date, and building a 3D homology model of this seven Kazal domains inhibitor based on the available 3D structure of a single Kazal-like domain would be very speculative. Although we do not have any information on the distribution of electrostatic charges on its surface, its antibacterial activity is likely to involve the inhibitory site rather than cationicity, considering its slightly acidic pI of 6.4 (**Table 1**).

#### 3.5.2 Chicken Cystatin

**Figure 6:**
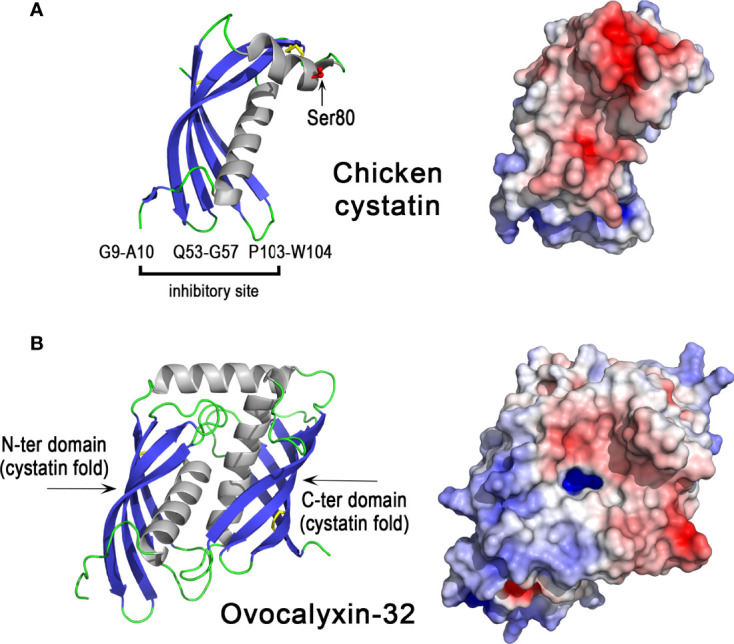
3D structures of egg antiproteases with cystatin fold, chicken cystatin and ovocalyxin-32. 3D structure of chicken cystatin **(A)** and chicken ovocalyxin-32 **(B).** The left panels correspond to the cartoon representation of 3D structure while the right panels show the color-coded electrostatic potential molecular surface. Color scheme ranges from red (negatively charged regions) to deep blue (positively charged regions). The figure was prepared using Pymol software (48) and APBS (Adaptive Poisson-Boltzmann Solver) plugin (49) for electrostatic calculations using X-ray atomic coordinates of 1CEW PDB file for chicken cystatin. The 3D structure of OCX-32 was modeled using the AlphaFold method and was retrieved in the AlphaFold protein structure database under accession number AF-Q90YI1-F1 (72, 73).

Chicken cystatin or chicken egg white cystatin was the first identified member of the cystatin superfamily of cysteine protease inhibitors (128, 129). Its orthologs are present in different animal classes including Mammalia, Aves, Reptilia, Amphibia, Actinopterygii (ray-finned fishes), but not in Chondrichthyes (cartilaginous fishes) nor in Cyclostomata (jawless fishes) (118). Family 2 cystatins including chicken cystatin contain two disulfide bonds (**Figure 6A**, left panel) and inhibit cysteine proteases such as papain and lysosomal cathepsins B, H, L. The inhibition mechanism of cystatins does not obey the “standard mechanism” of Kazal-like inhibitors described above (130). The inhibitory site of chicken cystatin includes the peptide bond between Gly9 and Ala10, the QLVSG variation sequence of the QVVAG consensus sequence (Gln53-Gly57) and the dipeptide Pro103-Trp104 (131). The cystatin fold corresponds to a long helix packed against a twisted five-stranded antiparallel β-sheet (described as a « hot dog fold »). The protease inhibitory site is formed by two hairpin loops (Gln53-Gly57 and Pro103-Trp104 regions) that together with the N-terminal end (Gly9-Ala10) form a wedge-shaped edge, which is highly complementary to the active site cleft of cathepsin-like cysteine proteases. These characteristics facilitate the formation of a high-affinity protease-cystatin complex, thereby preventing access of substrates to the protease active site. At the opposite side of the inhibitory site, the region 70 to 93 contains a short α-helix (77-85) in the X-ray structure (**Figure 6A**) but adopts an extended conformation in contact with the central β-sheet in the NMR structure i.e in solution (not shown). It is not known however, whether the conformational variability of this region, which contains one of the two disulfide bridges of chicken cystatin and a phosphorylated serine (Ser80), has consequences for its biological activities. With a value of 7.3, the pI of chicken cystatin is neutral (**Table 1**) and the distribution of electrostatic charges on the molecular surface of cystatin reveals large negatively charged areas (**Figure 6A**, right panel).

The broad antimicrobial spectrum of chicken cystatin is evidenced by its capacity to inhibit proteases from viruses, parasites and bacteria (132, 133). It has been shown to have antibacterial activity against *Porphyromonoas gingivalis* (132, 134), *E. coli* (135, 136) and various bacteria including *Acinetobacter lwoffii*, *Oligella* sp. and *P. aeruginosa* (136). Similar to ovoinhibitor, chicken cystatin is a protein constituent of the cuticle layer (36), and thus it could influence eggshell surface colonization by pathogens that secrete cysteine proteases. Chicken cystatin is the major protease inhibitor identified in eggshell. It is present at high concentration in the uterine fluid during the final phase of eggshell formation, and is among the 20 most abundant proteins of the eggshell (124). It is thus proposed to play a major role in the process of eggshell formation, although the importance of its protease inhibitory activity in this process remains to be explored. Indeed, besides cathepsin B that was identified with a relatively low abundance (90^th^ based on the emPAI value) (124), no additional cysteine protease has been identified in the chicken uterine fluid or eggshell proteome. Notably, recent findings revealed that mouse cystatin C binds glycosaminoglycans, including bone heparan sulfate, in a pH-dependent manner (137). Since the eggshell contains glycosaminoglycans including heparan sulfate (138, 139), the role of chicken cystatin in eggshell formation could involve binding to eggshell glycosaminoglycans/proteoglycans, by a mechanism independent of its protease inhibitory activity. However, the potential interaction of cystatin with glycosaminoglycans or the mineral phase of the eggshell remains to be confirmed.

#### 3.5.3 Ovocalyxin-32

Ovocalyxin-32 (OCX-32) is a 32 kDa protein that exhibits about 30% identity with human carboxypeptidase inhibitor latexin and human retinoic acid receptor-responder 1 (RARRES1) (29). It belongs to the protease inhibitor I47 (latexin) family (125). Members of this family, such as mouse and human latexin, adopt a cystatin-like fold similar to that found in chicken cystatin (**Figure 6A**). OCX-32 contains two cystatin-like units (**Figure 6B**) and is phosphorylated at serine residues 261 and 268 (129, 140). The distribution of electrostatic charges reveals two domains, one cationic domain (N-ter domain) and one rather acidic domain (C-ter domain) (**Figure 6B**, right panel), which may be involved in distinct interactions with eggshell components (other proteins, glycosaminoglycans, mineral phase). Recombinant OCX-32 is able to inhibit bovine carboxypeptidase (30), but a cysteine protease inhibitory activity of OCX-32 has not yet been evaluated. This phosphoprotein localizes to the outer palisade layer, the vertical crystal layer, and the cuticle of the eggshell and thus is predicted to mainly have a role during the terminal phase of eggshell formation (29); however, it is also relatively abundant in the egg perivitelline layer (vitelline membrane) (141, 142). OCX-32 is one of the most abundant constituents of the cuticle (143). Recombinant OCX-32 was reported to impair the growth of *B. subtilis* (30). The chromosomal location of the *OCX32*/*RARRES1* gene is highly conserved in a syntenous gene locus from fishes to mammals (47).

The exact role of OCX-32 in eggshell formation is not yet known but this gene has been genetically associated with eggshell strength and quality, as well as mammillary knob layer thickness (144–146). Its relative abundance in the uterine fluid increases during the terminal phase of eggshell formation (43^rd^ based on emPAI value) (124). Altogether, these data highlight the crucial function of OCX-32 in the formation of the eggshell but the underlying molecular mechanisms, and a potential link to its antiprotease activity remains unknown.

#### 3.5.4 Ovalbumin-Related Protein X

**Figure 7:**
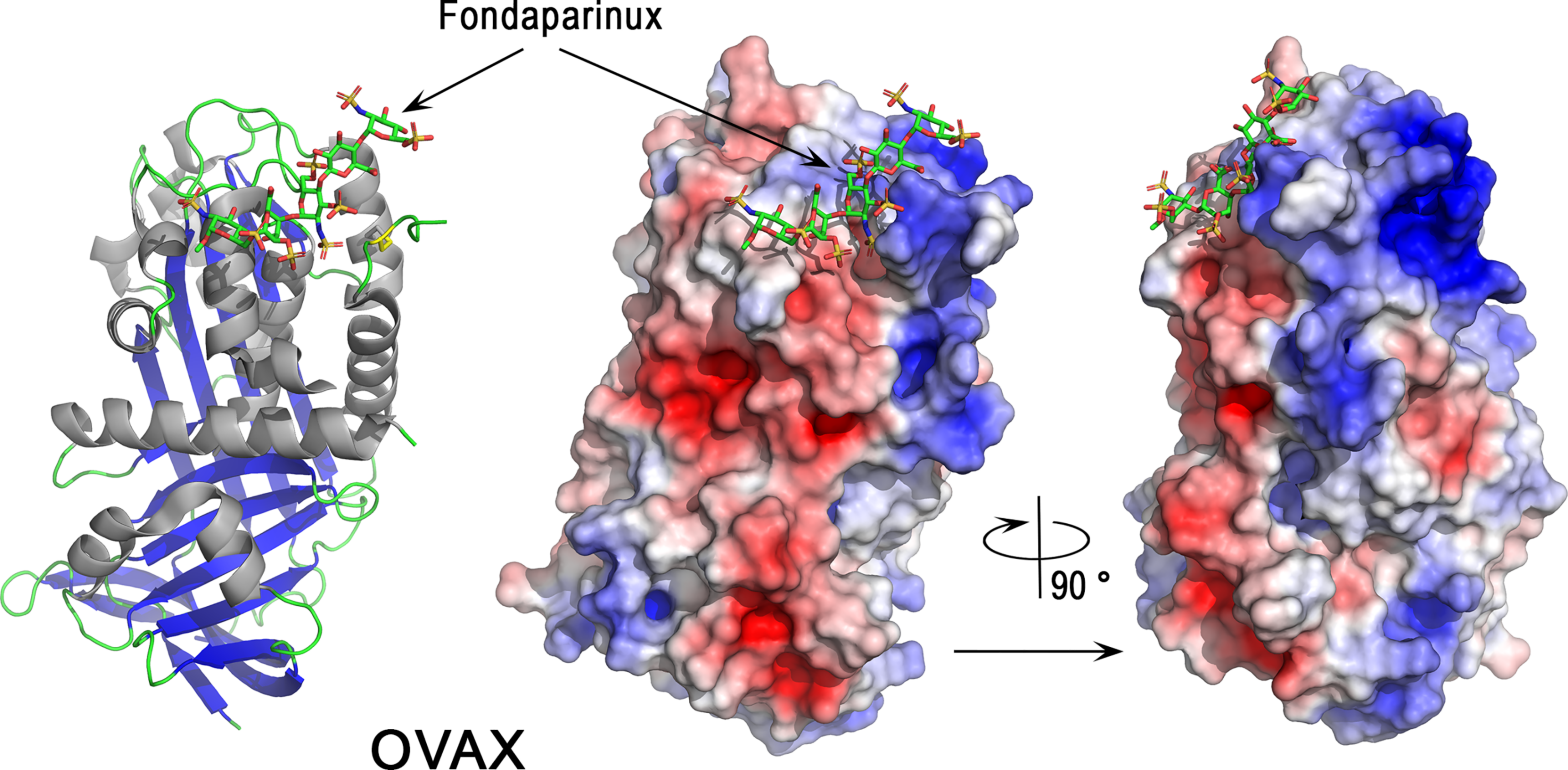
3D structure of the serpin ovalbumin-related protein X (OVAX). The left panel corresponds to the cartoon representation of 3D structure of chicken OVAX while the right panel shows the color-coded electrostatic potential molecular surface. Color scheme ranges from red (negatively charged regions) to deep blue (positively charged regions). Fondaparinux is shown in stick representation (carbon: green, oxygen: red, sulfur: orange, nitrogen: blue). The figure was prepared using Pymol software (48) and APBS (Adaptive Poisson-Boltzmann Solver) plugin (49) for electrostatic calculations using X-ray atomic coordinates of OVAX kindly provided by F. Coste (CBM, CNRS, UPR4301, Orléans, France) prior to the release of these data in the PDB under 7QRN accession code.

Ovalbumin-related protein X belongs to the serpin family (SERine Protease INhibitor, I4) and more precisely to the subgroup clade B serpins, also known as ov-serpins (125, 147). Serpins are a superfamily of proteins with similar structures. Many serpins are protease inhibitors that inhibit proteases *via* an unusual mechanism, in which they form an irreversible complex with their target protease, accompanied by a large conformational change in the inhibitor. The OVAX gene (SERPINB14C) co-localizes within a 46 kb locus with two paralogous genes, OVAY (ovalbumin-related protein Y) and ovalbumin, on chromosome 2 (148). The three paralogs ovalbumin, OVAX and OVAY seem to be bird-specific (149). According to the Ensembl database (March 2022), they have a common ancestor with a crocodilian gene (ENSCPRG00005003287) and possess orthologs with the human paralog (SERPINB3, SERPINB4), which suggests that independent duplications occurred in birds and in mammals from a common ancestor. This observation is, however, at variance with previous findings indicating that the ovalbumin, OVAX and OVAY genes arose from SERPINB12 by duplication events after the split between birds and mammals (147). Similar to ovalbumin, OVAX lacks inhibitory activity against serine proteases (150) and little is known regarding its physiological functions. However, OVAX possesses antibacterial activity against *Listeria monocytogenes* and *S.* Enteritidis, and this activity is blocked in the presence of heparin glycosaminoglycan (150). A cationic domain present in OVAX, but not in ovalbumin or in OVAY structures (151), is likely to be responsible for this antibacterial activity, as it could explain the interaction of OVAX with the negatively charged bacterial cell wall. X-ray structural analysis of OVAX complexed to Fondaparinux (a synthetic pentasaccharide derived from heparin) confirms the ability of OVAX to bind glycosaminoglycans (**Figure 7**) in a region that is close to the cationic domain that was initially predicted to bind heparin (150). This pentasaccharide interacts *via* a network of hydrogen bonds involving Gly57, Asn58, Glu64 of OVAX and a salt bridge formed between the Arg56 side chain with a sulfate group of the pentasaccharide (**Figure 7**). The Fondaparinux binding site of OVAX contains both negatively and positively charged areas at the top of the molecule as depicted (**Figure 7**).

The gene expression of OVAX is oviduct-specific and induced by estrogen (152). Although OVAX is mainly expressed in the magnum (responsible for egg white formation) (151), it was identified in the eggshell and its relative abundance (emPAI) was elevated in uterine fluid during the terminal phase of eggshell formation (45^th^ based on EmPAI value) (124). The role of OVAX in eggshell formation has not been explored yet but it is noteworthy that its paralog ovalbumin modifies calcium carbonate crystallization and potentially plays a role in ACC stabilization and eggshell calcification (40, 153, 154). This mechanism of action of ovalbumin possibly involves acidic amino acids that would bind Ca^2+^ cations. The analysis of OVAX structure also reveals the presence of several negatively charged regions (**Figure 7**, right panel). This observation, together with the capacity of OVAX to bind heparin and possibly eggshell glycosaminoglycans, should stimulate studies to further explore its role in eggshell mineralization.

#### 3.5.5 Ovostatin

Ovostatin belongs to the alpha2-macroglobulin family, whose members undergo a unique interaction with proteases (cysteine, serine, aspartic proteases and metalloproteases). Ovostatin is a tetramer of similar subunits, of which two pairs are bound by disulfide bonds (dimers) and then assemble non-covalently. The inhibitory mechanism of macroglobulins begins with the cleavage of the macroglobulin “bait” region by the protease, which induces a massive conformational change of the inhibitor, while entrapping the targeted protease according to a “venus flytrap” mechanism (155, 156). Another specificity of the macroglobulin-protease complex is the formation of a covalent thiol-ester bond between the protease and the inhibitor, while the active site of the enzyme remains accessible. Consequently, small peptidic substrates can still be hydrolyzed by the complexed protease, in contrast to protein substrates (or antibodies) that cannot reach the protease active site because of steric hindrance (157). Ovostatin is rather acidic (pI = 5.6, **Table 1**). This protease inhibitor, also termed ovomacroglobulin, has been shown to inhibit the growth of protease-producting bacteria such as *Serratia marcescens* and *P. aeruginosa* (158). These data suggest that the antibacterial effect of ovostatin against bacteria involves the inhibitory site. In addition, ovostatin may participate in host immunity by binding to host antimicrobial compounds, protecting them from degradation by microbial proteases (159). The exact role of ovostatin in the process of eggshell mineralization is not clear, and its interaction with the eggshell mineral has not yet been studied. This protein was identified in low abundance at the early stages of eggshell formation (i.e at 5, 6 or 7 h post-ovulation) (160) and may regulate proteolytic activities in the uterine fluid (protein processing and/or degradation). Unfortunately, the 3D structure of ovostatin is not available and the structure model available in the Alphafold database (72, 73) is not suitable for further structural analysis.

### 3.6 Chelators and Chelator-Binding Proteins: Avidin, Ovotransferrin, Ex-FABP

#### 3.6.1 Avidin

**Figure 8:**
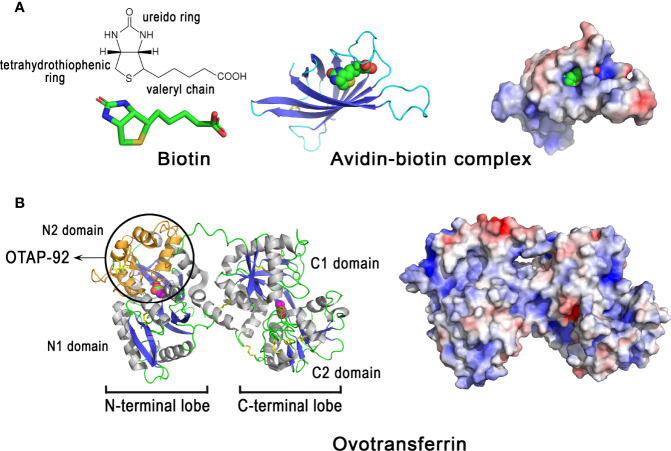
3D structure of egg proteins with chelating activities: avidin and ovotransferrin. 3D structure of chicken avidin **(A)** and chicken ovotransferrin **(B).** The left panels correspond to the cartoon representation of 3D structure while the right panels illustrate the color-coded electrostatic potential molecular surface. Color scheme ranges from red (negatively charged regions) to deep blue (positively charged regions). Avidin **(A)** is complexed to the vitamin biotin through numerous interactions including H-bonds and hydrophobic interactions. Ovotransferrin **(B)** possesses two lobes, each containing two domains. Each lobe binds a Fe^3+^ ion and a carbonate ion. Location of the antibacterial peptide OTAP-92 is also shown. The figure was prepared using Pymol software (48) and APBS (Adaptive Poisson-Boltzmann Solver) plugin (49) for electrostatic calculations using atomic coordinates of 2AVI and 1N04 PDB file for avidin-biotin complex and ovotransferrin, respectively.

Avidin belongs to a family of homologous proteins (avidin gene family) encoded within a gene locus on the sex chromosome Z (161). Avidin-like genes are present in most vertebrates except mammals, which suggests that avidin has been lost in the mammalian branch (149). Chicken avidin is a cationic homotetrameric glycoprotein (≈ 67 kDa, pI = 10.0, **Table 1**) with an exceptionally high affinity for biotin. The dissociation constant of the avidin-biotin complex is about 10^−15^ M (162). Although detected in avian eggshell (**Table 1**), avidin is enriched in the egg white, where it constitutes 0.05% of the total albumin protein (78). Oviductal avidin gene expression can be induced by progesterone or bacterial infection (163, 164). Avidin monomer (**Figure 8**, left panel) forms a β-barrel made up of eight right-handed twisted antiparallel strands. Although the pI of avidin is very cationic, the charges are evenly distributed on the surface of the molecule (**Figure 8**, right panel). The active tetramer (not shown) is stabilized by an extended network of intermonomer hydrogen bonds, in addition to four salt bridges. The exceptional high affinity of avidin for biotin is due to a large number of hydrogen bonds between both partners (e.g, 5 H-bonds are formed between polar residues of avidin with the ureido ring of biotin) as well as hydrophobic interactions involving the tetrahydrothiophenic ring and the valeryl chain of biotin with Phe and Trp aromatic residues lining the cavity (165). The half-life of the complex is about 200 days at pH 7.0 (166), indicating that the avidin-biotin binding complex is virtually irreversible. This protein is proposed to inhibit the growth of biotin-requiring microorganisms in eggs (167). Under experimental conditions, avidin slightly inhibits the growth of an *E. coli* mutant dependent on exogenous biotin (168). It can also bind to various Gram-negative and Gram-positive bacteria, including *E. coli*, *Klebsiella pneumoniae*, *S. marcescens*, *P. aeruginosa*, *S. aureus* and *Staphylococcus epidermis*, independent of its biotin-binding properties (169). The interaction of avidin with the *E. coli* cell wall is mediated by the porin protein (OmpF/OmpC) of the outer membrane (169).

#### 3.6.2 Ovotransferrin

Ovotransferrin, previously known as conalbumin, is a glycoprotein of about 78-80 kDa, with an isoelectric point of 7.3 (**Table 1**) and metal ion-binding properties. It is highly abundant in egg white (≈ 13 mg/mL), accounting for about 13% of total protein. Ovotransferrin is mainly synthesized by the magnum (and white isthmus), but its gene expression is also detected, at lower levels, in red isthmus and uterus where mineralization of the eggshell occurs (23). In eggshell, the protein is mainly detected in eggshell membranes and in mammillae (23). Ovotransferrin is similar to serum transferrin, as both proteins are encoded by the same gene (TF), but they differ in glycosylation pattern. The synthesis of ovotransferrin in the oviduct is regulated by estrogen and progesterone (170). The 686 amino acid residue polypeptide chain of ovotransferrin forms two lobes referred to as N- and C-terminal lobes,connected by a nine-residue α-helix (**Figure 8B**, left panel). Each lobe consists of two α/β fold subdomains of about 160 amino acids that are connected by a two stranded β-sheet. Each lobe contains one iron-binding site, located in a cleft between the subdomains of each lobe. Fe3^+^ ion binds reversibly with high affinity along with a carbonate $\left({\text{CO}}_{3}^{2-}\right)$ anion. Six coordination bonds are formed with iron, four by amino acid ligands and two by the carbonate ion. X-ray studies of the apo- and di-ferric forms of ovotransferrin reveal that both lobes have a closed conformation when iron is bound, while they are open in the apo-form (171). This implies domain movement in order to bind or release Fe3^+^. The binding of iron to ovotransferrin involves the side-chains of four amino acid residues (one Asp and one His in domain 1, two Tyr in domain 2), which are conserved in both lobes, as well as two oxygen atoms of carbonate ion (172). The N and C lobes have apparent binding constants for ferric ions of 1.5 × 10^-14^ M and 1.5 × 10^-18^ M, respectively, at pH 7 (173). Positive and negative charges are evenly distributed on the surface of the molecule (**Figure 8B**, right panel).

Ovotransferrin exhibits antibacterial properties that occur either *via* an indirect mechanism (its ability to chelate iron, an important nutrient for bacterial growth), or directly through its ability to interact with the bacterial cell surface and to induce membrane perturbation (174) or membrane permeation (175). Ovotransferrin has a bacteriostatic effect on *S. enterica* growth in a rich medium (BHI), that is in part inhibited when the protein is in holo form (iron saturated); however, bactericidal activity is observed against the same bacteria in phosphate buffered saline without Ca^2+^/Mg^2+^ independent of the iron status of ovotransferrin (apo or holo) (176). It has been suggested that ovotransferrin can chelate divalent ions present on the outer membrane of Gram-negative bacteria and thus induce membrane perturbation, similar to other members of the transferrin family (174). Ovotransferrin can also permeate the outer membrane of *E. coli* to reach the inner membrane and induce selective permeation of ions (175). Bactericidal activities were reported for ovotransferrin against *S. aureus* and *B. cereus* (177, 178). Interestingly, acidic proteolysis of ovotransferrin produces a cationic fragment, named OTAP-92 (**Figure 8B**, left panel), which consists of a 92 amino acid sequence located in the N-terminal lobe (Leu109 to Asp200), that possesses a bactericidal effect against *S. aureus* and *E. coli* (178, 179). The two helix-sheet motifs of the corresponding peptide in the conformational context of ovotransferrin is reminiscent of the insect defensin fold, thereby providing a putative structural basis for its bactericidal activity (178, 179).

It has been demonstrated that, depending on the pH, the mechanisms associated with iron deprivation and/or membrane perturbation are involved in the bactericidal properties of ovotransferrin against *B. cereus* (177). Interestingly, ovotransferrin exhibits an antiviral activity against the Marek’s disease virus (176).

Ovotransferrin may have dual functions in the eggshell context, with physiological roles not only associated with antimicrobial defense, but also with the eggshell mineralization process. *In vitro* experiments indeed demonstrated that purified ovotransferrin is able to modify the size and morphology of calcite crystals (23). These biomineralizing properties might be related to its location at and around the site of eggshell mineralization, and possibly to its carbonate-binding properties.

Ovotransferrin belongs to the transferrin family, a group of homologous proteins found in vertebrates and invertebrates (180). Vertebrate transferrin family members include, among others, serum transferrin, melanotransferrin and mammalian lactoferrin. The two homologous lobes of these proteins likely resulted from an ancient gene duplication event, prior to the protosome/deuterostome split (> 670 million years ago), which was then followed by another duplication, leading to serum transferrin and melanotransferrin ancestral genes (180). In contrast, lactoferrin seems to have appeared relatively recently. Published phylogenetic trees indeed suggest that mammalian serum transferrin and lactoferrin arose by duplication after the bird-mammal split (171, 180, 181). In mammals, serum transferrin is involved in iron homeostasis and insures the transport of iron in physiological fluids (e.g. blood), while lactoferrin is instead involved in innate immunity in bodily secretions (e.g. milk, tears…) (180). In birds, egg white ovotransferrin and serum transferrin are encoded by the same gene, and thus correspond to the mammalian serum transferrin (180). Hence, the ovotransferrin gene product and its glycosylation pattern may have evolved towards a dual function, possessing roles in both iron homeostasis and transport, and immunity. More particularly in avian eggshell, an innate immune role for ovotransferrin may be more complex than being only an antimicrobial, since it is potentially involved in eggshell mineralization, and therefore also making an indirect contribution to physical barrier defense of the egg contents.

#### 3.6.3 Extracellular Fatty Acid Binding Protein

Ex-FABP (extracellular fatty acid binding protein), previously known as Ch21 protein, is a protein belonging to the lipocalin family, whose members bind fatty acids and various ligands including small hydrophobic molecules (steroids, bilins, retinoids…) and proteins. It has a predicted molecular weight of 18 kDa and a theoretical pI of 6.3 (**Table 1**). Ex-FABP is present in chicken eggshell, along with other lipocalins, such as ovoglycoprotein/α-1-acid glycoprotein, Cal-γ/chondrogenesis-associated lipocalin, apolipoprotein D (35). Interestingly, Ex-FABP is developmentally regulated in chicken endochondral bone formation (182), which suggests that it may have a role in biomineralization processes. This protein is a siderophore-binding protein similar to mammalian siderocalin (Scn), a lipocalin involved in the sequestration of various ferric siderophores, including enterobactin, parabactin and bacillibactin (183). Siderophores are small high-affinity iron-chelating molecules, especially produced by bacteria upon iron restriction to scavenge and internalize extracellular iron ions required for their growth. Interaction of siderophores with ferric iron is characterized by both an exceptional stability and affinity, with K_D_ values reaching 10^-30^ M or even greater in some cases (184). Indeed, the affinity of siderophores for iron is so strong that they can acquire the iron bound to ovotransferrin or transferrins (174). Siderophore-binding proteins can thus counter this bacterial siderophore strategy. Ex-FABP was demonstrated to specifically and tightly bind ferric complexes of enterobactin (K_D_ = 0.22 nM), parabactin (K_D_ = 42 nM), bacillibactin (K_D_ = 14 nM) and monoglucosylated enterobactin (K_D_ = 0.07 nM) (185).

**Figure 9:**
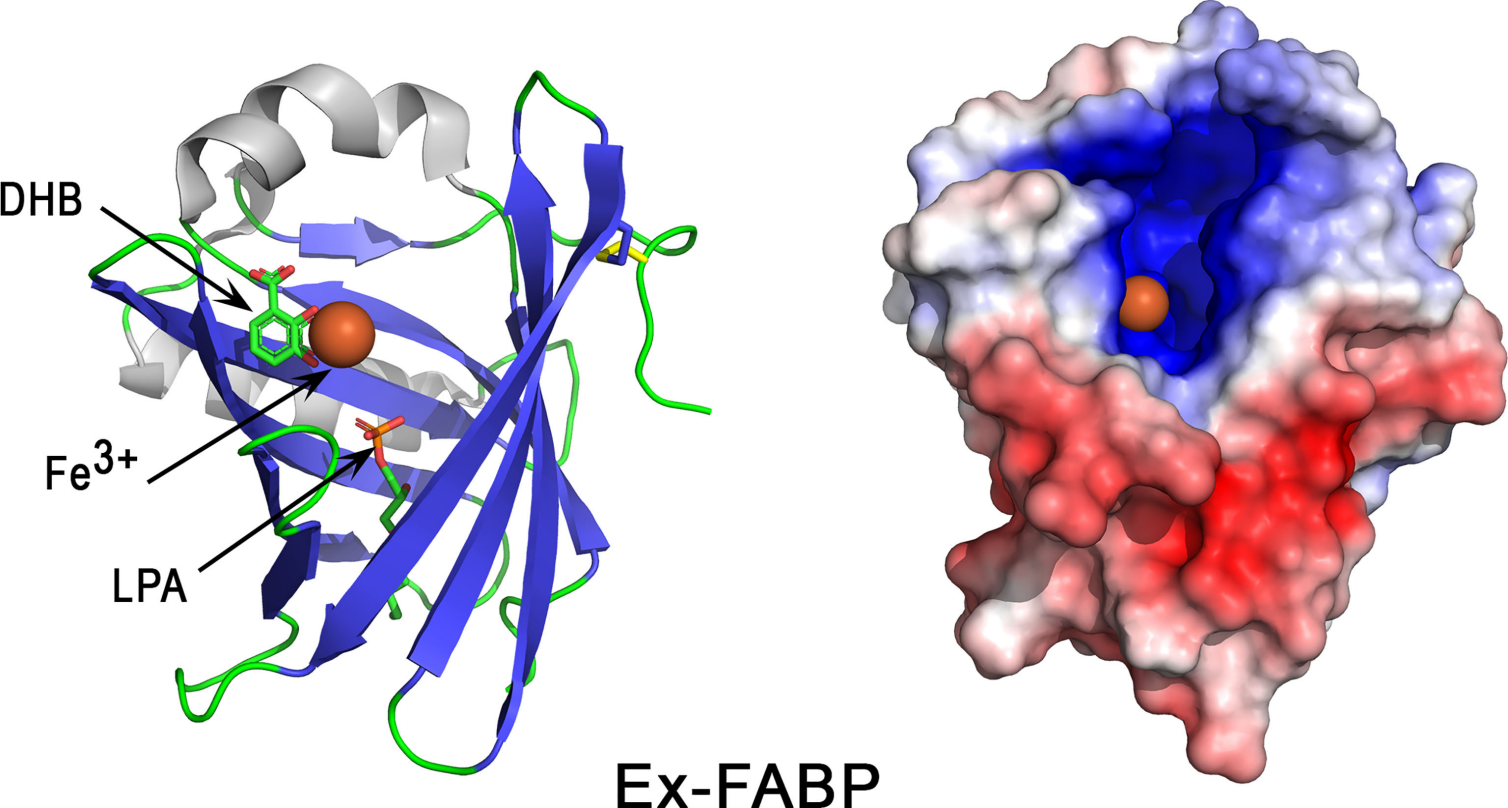
3D structure of the iron-chelator Ex-FABP. The left panel corresponds to the cartoon representation of 3D structure of chicken Ex-FABP while the right panel shows the color-coded electrostatic potential molecular surface. Color scheme ranges from red (negatively charged regions) to deep blue (positively charged regions). The figure shows an Fe^3+^ ion (colored orange)-dihydoxybenzoate complex bound in one of the three subcavities of Ex-FABP calyx. Dihydroxybenzoate (DHB) and lysophosphatidic acid (LPA) are shown in stick representation. The figure was prepared using Pymol software (48) and APBS (Adaptive Poisson-Boltzmann Solver) plugin (49) for electrostatic calculations using 3SAO PDB file for chicken Ex-FABP structure.

The 3D structure of Ex-FABP consists of an eight-stranded antiparallel β-barrel together with accessory helical elements (**Figure 9**, left panel). The cavity formed by the β-barrel structure corresponds to the ligand binding site, namely a cup-like cavity, the “calyx”. Similar to Scn, but contrasting with most lipocalins, the calyx of Ex-FABP has a net positive charge attributed to three basic residues (Lys82, Arg101, and Arg112) (185). These residues are presumed to be crucial for the siderophore-binding properties of Ex-FABP since the side chains of corresponding residues in Scn (Arg81, Lys125, and Lys134) insert between the three catecholate rings of ferric enterobactin to form a complex stabilized by ionic and cation-π interactions (183). Remarkably, the molecular surface (colored according to electrostatic potential values) shows a highly basic hotspot restricted to the calyx region (**Figure 9**, right panel), while the remaining surface is neutral or very acidic (**Figure 9**, right panel), in accordance with the global acidic pI value of Ex-FABP (**Table 1**).

Ex-FABP exhibits bacteriostatic activities *via* its ability to bind ferric siderophores. Such activities have been reported *in vitro* against various bacteria, including *E. coli* (185, 186), *B. subtilis* (185) and a salmochelin-deficient *S.* Enteritidis mutant (187). The growth inhibition of *E. coli* is abolished when native Ex-FABP is replaced with a double mutant Arg101Ala/Arg112Ala or by addition of stochiometric amounts of iron (185), which thus demonstrates that the bacteriostatic activity of Ex-FABP is mediated through iron-siderophore complex sequestration. No inhibiting activity was observed against *P. aeruginosa* (185) or wild-type *S.* Enteritidis (187), possibly because these bacteria secrete siderophores that are not sequestered by Ex-FABP, like salmochelin in *Salmonella*.

### 3.7 Other Antimicrobial Proteins

#### 3.7.1 Beta-Microseminoprotein-Like (MSMB3)

Beta-microseminoproteins (MSMBs) are non-glycosylated disulfide-rich small proteins that have been identified in many animal species. MSMB genes are widely distributed among vertebrates (188). Three chicken paralogues (MSMB1, MSMB2, MSMB3), whose genes are localized on chromosome 6, have been described (189). Orthologs of these three genes are found in several bird species and, interestingly, the degree of conservation between avian MSMB3 orthologs is higher than that observed between avian MSMB1 or MSMB2 orthologs (188). Of note, murine MSMB protein is more similar to avian MSMB2 than MSMB1 and MSMB3 (188). Given the apparent oviduct-specific expression of chicken MSMB3 (189), it is thought that the biological function(s) of avian MSMB3 might have functionally diverged from that of other vertebrate or mammalian MSMBs, towards a specific role in reproduction. The phylogeny and evolutionary description of MSMB gene(s) within reptiles remains to be investigated.

**Figure 10:**
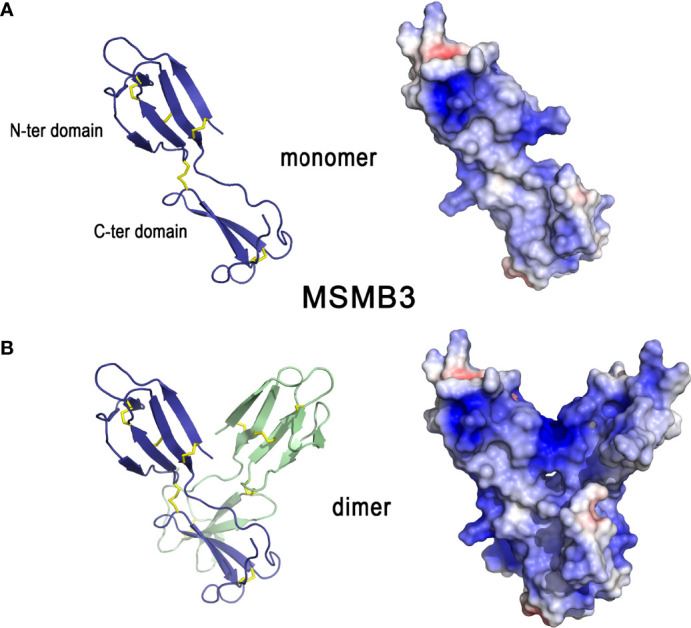
3D structures of β-microseminoprotein-like MSMB3. 3D structure of chicken MSMB3 monomer **(A)** and dimer **(B)**. The left panels correspond to the cartoon representation of 3D structure while the right panels show the color-coded electrostatic potential molecular surface. Color scheme ranges from red (negatively charged regions) to deep blue (positively charged regions). The figure was prepared using Pymol software (48) and APBS (Adaptive Poisson-Boltzmann Solver) plugin (49) for electrostatic calculations using atomic coordinates of 6RWC PDB file.

The X-ray crystal structure of MSMB3 (189) shows that it is composed of a N-terminal domain linked to a C-terminal domain by a linker peptide (**Figure 10A**, left panel). The N-terminal domain has a four-stranded antiparallel β-sheet arranged in a Greek key motif whereas the C-terminal domain has one antiparallel β-sheet composed of two strands. Both domains are linked by a disulfide bridge that reduces the flexibility of each monomer in the linker region. Four other disulfide bonds stabilize the whole monomer. Similar to its human and porcine counterparts, chicken MSMB3 can be found as a homodimer in solution (**Figure 10B**). However, unlike human and porcine MSMB3 dimers in which each monomer associates in an edge-to-edge mode, MSMB3 monomers associate in a different pattern, forming most interactions between their respective C-terminal domains (**Figure 10B**) (189). It is not yet known whether the biologically active species is the monomer, the dimer, or both forms. The physiological function of these proteins are not known, but MSMB3 purified from egg white displays antibacterial activities against *L. monocytogenes* and *S.* Enteritidis (38). It is noteworthy that chicken MSMB1 and MSMB2 remain uncharacterized because they are not yet available as purified proteins (189). Similar to OVAX, MSMB3 antibacterial activity is blocked by heparin, which suggests that the MSMB3 heparin-binding site is important for the interaction with bacteria. Compared to MSMB1 and MSMB2, MSMB3 is highly cationic (pI=10.4, **Table 1** **,** **Figure 10B**, right panel), which might partly explain its antibacterial activity (189). MSMB3 has been identified in shell membranes (33, 34) consistent with its expression by the white isthmus segment of the oviduct (189). The crystal structure of MSMB3 includes three sulfate ions. Interestingly, although this feature is likely due to the buffer used for crystallization, it illustrates the affinity of MSMB3 for anions. Altogether, these findings support a potential role for MSMB3 in eggshell mineralization, possibly through interaction with eggshell glycosaminoglycans or proteoglycans such as the heavily phosphorylated and abundant dermatan sulfate proteoglycan OC-116. Its role during eggshell mineralization is likely to occur during the initial phase, considering its presence in eggshell membrane (**Table 1**).

#### 3.7.2 Pleiotrophin

Pleiotrophin is a cationic glycosaminoglycan-binding growth factor/cytokine involved in many biological functions including neural development, angiogenesis, inflammation/injury, bone development, adipocyte differentiation, and mammary epithelial cell differentiation (190). Orthologs of this protein are found in all vertebrates. In chicken eggshell, pleiotrophin is detected in the calcified layer (**Table 1**). In the oviduct, the pleiotrophin gene (PTN) is expressed in all oviductal segments, but higher levels of PTN mRNA are detected in the isthmus and the uterine segments (191). Its expression in the oviduct is regulated by estrogen (191).

**Figure 11:**
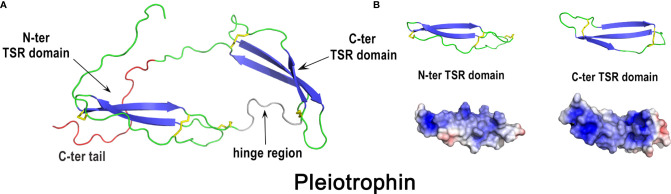
3D structure of pleiotrophin. The left panel **(A)** corresponds to the cartoon representation of chicken pleiotrophin 3D structure while the right panel **(B)** illustrates the color-coded electrostatic potential molecular surface of N-ter and C-ter domains. Color scheme ranges from red (negatively charged regions) to deep blue (positively charged regions). Pleiotrophin has two thrombospondin type-1 repeat domains (TSR), one with a two-stranded β-sheet (N-ter domain) and the second with a three-stranded β-sheet (C-ter domain) linked by a flexible hinge region. Both TSR domains are flanked by unstructured termini. The structure of chicken pleiotrophin was predicted by comparative modeling using SwissModel server (swissmodel.expasy.org) and human pleiotrophin (PDB code 2N6F) as a template. The figure was prepared using Pymol software (48) and APBS (Adaptive Poisson-Boltzmann Solver) plugin (49) for electrostatic calculations.

The amino acid sequence of pleiotrophin is highly conserved (> 90% identity) between chicken and mammals (192, 193), which thus suggests broad evolutionary conservation of its structure and biological functions between birds and mammals. The structural model of chicken pleiotrophin (**Figure 11A**), based on human pleiotrophin (194), shows that the protein contains two thrombospondin type-1 repeat domains (TSR), one with a two-stranded β-sheet (N-ter domain) and the second with a three-stranded β-sheet (C-ter domain). Both domains are linked by a flexible hinge region and flanked by unstructured termini. NMR studies indicate that these two domains appear to be independent of each other and consequently, the flexible hinge region does not have well-defined interdomain orientation. Many different conformations seem to coexist for the whole molecule in solution. Pleiotrophin exhibits highly positively charged surfaces (pI = 11.0, **Table 1** **,** **Figure 11B**, right panel). Interestingly, stretches of lysine residues at the N- and C-termini as well, as basic clusters within both these domains, are strictly conserved compared to human pleiotrophin. This observation suggests that positively charged regions of both domains could interact with negatively charged structures of bacterial cell walls and/or eggshell glycosaminoglycan units. The C-terminal tail and the hinge region, although non-structured, have been shown by NMR titration studies to interact synergistically with TSR domains to bind chondroitin sulfate A (194), in agreement with its high basic residue content. The N- and C-terminal domains of pleiotrophin have three and two disulfide bonds, respectively. Human pleiotrophin is known to interact with relative high affinity to some glycosaminoglycans like chondroitin sulfates (K_D_ of 5-251 nM) (195) or heparin (K_D_ of 4-460 nM) (196), and to the proteoglycan syndecan 3 (K_D_ of 6 nM) (193). The C-terminal domain and the hinge region are the major binding sites for chondroitin sulfates (194) and for heparin (197, 198). Interestingly, the C-terminal tail of pleiotrophin contributes to the affinity of pleiotrophin for chondroitin sulfate A, but not for the highly sulfated chondroitin sulfate E (194).

Chicken pleiotrophin possesses heparin affinity and antibacterial properties (38), like human pleiotrophin (199), and may therefore participate in egg antimicrobial protection. Although human pleiotrophin seems to play a role in bone development and repair (190, 200), the implication of chicken pleiotrophin in eggshell biomineralization remains to be demonstrated.

## 4 Phylogeny and Comparative Biology of Chicken Eggshell Antimicrobial Proteins and Peptides in Avian and Non-Avian Reptilian Species

The relatively narrow taxonomic restriction of the hard-shelled egg to Archosauria (Crocodilia, Aves), suggests that insight can be gained from phylogenetic analysis of the AMPPs discussed in this review. This section comprises a summarized phylogeny of chicken eggshell AMPPs to highlight species specificities of identified molecules (4.1) followed by an integrated comparison of these chicken AMPPs with the AMPPs identified in bird (4.2) and crocodile (4.3) eggshell proteomes. The main interest of such a comparative work is to point out similarities but also any potential differences that may reveal an adaptation of species during evolution to shape and optimize the eggshell AMPP constituents to provide improved egg protection against environmental pathogens.

### 4.1 Phylogeny of Chicken Eggshell AMPPs

Phylogenetic and structural data provided in previous sections and from the work of Le Roy et al. (47) and Da Silva et al. (149) reveal that most chicken eggshell AMPPs described in this review (lysozyme C, lysozyme G, ovostatin, ovotransferrin, VMO1, avidin, cystatin, Ex-FABP, OCX-32) are widely distributed in vertebrates, while a smaller subset are restricted to narrower clades such as tetrapods (TENP), amniotes (OCX-36), archosaurs (OC-17) or birds (AvBD11, OVAX, OvoDA1). Interestingly, it is noteworthy that some genes seem to have been lost in mammals (avidin) or in therian mammals (OCX-36) (81). Among eggshell AMPPs, OCX-36 is remarkable since its related gene has been found only in shelled-egg-laying species, including sauropsids and the monotreme mammal platypus. However, in spite of such a specific distribution, its role in eggshell formation remains unclear. OC-17 is also another notable component of chicken eggshell for several reasons. This C-type lectin is eggshell-specific, highly abundant in chicken eggshell, restricted to the clade of Archosauria, and it possesses dual functions with both antimicrobial and biomineralizing properties. Strikingly, proteins possessing the C-type lectin domain are present in the calcium carbonate biomineralized structures of evolutionarily distant species such as shell-forming mollusks (55), suggesting a generic role for this fold in biomineralization and possibly also in antimicrobial protection.

### 4.2 AMPPs in the Eggshell of Other Avian Species

Large differences in the SDS-PAGE protein profiles of the eggshell organic matrix are observed in different avian species (201), which suggests that the eggshell protein composition, in terms of protein types and/or relative abundances, is not strictly conserved across birds. The protein composition of chicken eggshell matrix has been exhaustively investigated using proteomic approaches since the first eggshell matrix proteome was released in 2006 (35). More recently, eggshell matrix proteomes of five additional avian species have been published, including turkey (202), quail (203), zebra finch (204), duck (205) and Guinea fowl (19). The complexity of the organic matrix of avian eggshell is evidenced by the large number of proteins identified in all available proteomes (from 149 in Guinea fowl to several hundreds in the other species). Among them, a set of 54 proteins are common in chicken, turkey, quail, zebra finch and Guinea fowl eggshell proteomes (19), and 64 are shared between chicken, turkey, quail, zebra finch and duck (205). Integration of available proteomes revealed that several chicken eggshell AMPPs are also identified in the eggshell of all species studied (206). Notably, this common set of eggshell AMPPs include OCX-36, lysozyme C, three chelators (ovotransferrin, Ex-FABP, avidin) and two protease inhibitors (ovoinhibitor, cystatin). Interestingly, OCX-36 is one of the most abundant constituents of the chicken, turkey, quail and zebra finch eggshell proteomes (202–204). It is noteworthy that some AMPPs detected in chicken eggshell (AvBD9, AvBD10, β-microseminoprotein-like MSMB3) have not yet been identified in the eggshell proteome of other avian species. Conversely, some potential AMPPs, not identified in chicken eggshell proteomes to date, are present in the eggshell of other species: ovodefensin B1 in Guinea fowl (19), AvBD12 in duck (205), LBP in turkey and zebra finch (202, 204), NK-lysin in turkey (202), XCA-1 (a paralogous C-type-lectin to OC-17) in zebra finch and Guinea fowl (19, 204). Regarding the C-type lectins, XCA-1 has been identified in the eggshell matrix of several ratite species (including ostrich, emu and rhea) in addition to XCA-2 (the OC-17 ortholog) (44, 45). Goose eggshell matrix also contains ansocalcin (belonging to the XCA-1 group) which was demonstrated to possess antimicrobial and CaCO_3_ biomineralization properties *in vitro* (31, 207, 208). As an important point, it should be mentioned that similarities and specificities regarding eggshell matrix proteomes must be carefully interpreted since the quality and annotation of genomic databases are critical limiting factors in the analysis of proteomic data.

### 4.3 AMPPs in the Eggshell of Non-Avian Reptilian Species

In comparison to avian species, proteomic data on non-avian reptile eggshells are scarce. To our knowledge, only one eggshell proteome, obtained from Siamese crocodile (*Crocodylus siamensis*) is available (209). Substantial differences were reported in terms of diversity and quantity of identified proteins. Of particular interest, some homologs of avian “eggshell-enriched” proteins such as ovocleidins (including OC-17) and ovocalyxins (including OCX-32) could not be identified in this study, despite the presence of these genes in the crocodile genome. Crocodile eggshell, however, contains several potential AMPPs similar to those identified in avian eggshells, especially homologs of ovoinhibitor (XP_019390939.1), ovostatin (XP_019371617.1), ovotransferrin (CAK18230.1), and LBP/BPI/PLUNC family members including homologs of TENP (XP_019395327.1), OCX-36 (XP_019395043.1), and BPIFCB (XP_019400352.1). All these eggshell proteins might contribute to the innate immune protection of crocodile eggs. Interestingly, another work revealed the existence of an abundant defensin-like peptide, named pelovaterin, in the eggshell of the Chinese softshell turtle (*Pelodiscus sinensis*) (94). In addition to its antibacterial activities (*P. aeruginosa, Proteus vulgaris*), this cationic cysteine-rich antimicrobial molecule is able to induce and stabilize the formation of a metastable vaterite phase *in vitro*, presumably *via* a mechanism involving self-aggregation of the peptide in the form of micellar nanospheres (94). Thus, this turtle eggshell peptide might have dual functions with both immune protection and biomineralizing properties.

## 5 Conclusion

**Figure 12:**
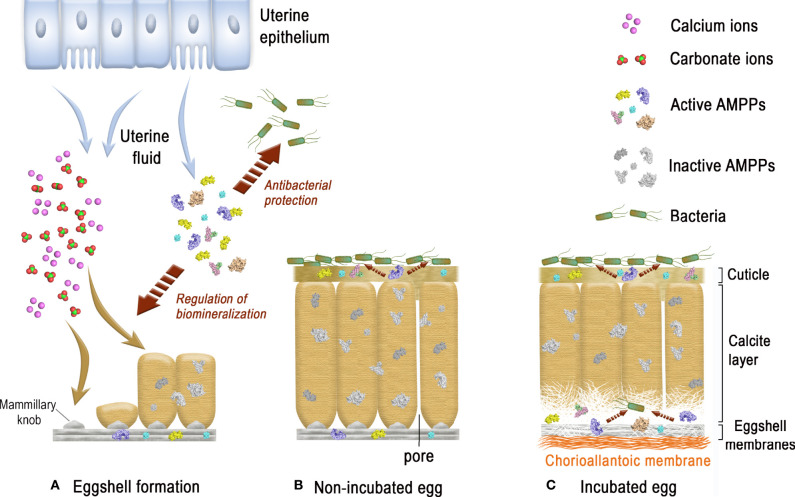
Graphical representation of the innate immune functions of eggshell AMPPs in eggshell mineralization and antibacterial protection. **(A)** During the formation of the eggshell in the uterus, ions of the mineral phase (calcium, carbonate ions) and proteins (including AMPPs) are secreted by the uterine epithelial cells into the extracellular milieu (uterine fluid). Some AMPPs can interact with ions and participate in the stabilization of ACC and/or regulate the growth of calcite crystals. These AMPPs become progressively embedded in the eggshell mineral during the mineralization process and are assumed to be inactive once immobilized. If bacterial contamination of the uterine fluid occurs during eggshell formation, the presence of soluble AMPPs can provide an antimicrobial defense to keep the interior of the egg free from pathogens. **(B)** Once the egg is laid, the eggshell exterior is exposed to environmental bacterial species that colonize the egg cuticle surface. In contrast to AMPPs embedded in the calcite layer, surface-exposed cuticle AMPPs may directly interact with bacteria and modulate the eggshell microbiome. **(C)** During egg incubation, the embryo-derived chorioallantoic membrane progressively develops at the inner surface of the eggshell membranes and creates a local acidification that dissolves the mammillary layer mineral to release calcium and carbonate ions required for embryonic bone calcification, and also to liberate the numerous mineral-associated proteins. Once solubilized, eggshell AMPPs are thought to recover their biological activity and locally reinforce innate immune protection to resist bacterial contamination following the penetration of pathogens through eggshell pores or microcracks. (Original artwork by TM, NG and HDF).

The avian eggshell allows gaseous exchange and provides calcium to the developing embryo while ensuring physical and antimicrobial protection. This calcitic structure consists of 95% calcium carbonate and 3.5% proteins, whose kinetics of deposition/secretion in the uterus of the laying hen is temporally controlled during eggshell formation. The eggshell proteins that become embedded in the mineral phase play a major role in defining the final eggshell ultrastructure. Interestingly, at least twenty of these proteins are also antimicrobials that may be classified into at least six families of antimicrobial proteins (lectins, BPI/LBP/PLUNC proteins, defensins, antiproteases, muramidases, chelators,…). The mechanism by which eggshell matrix proteins could act both in the biomineralization process and provide innate immune protection is not yet understood. In addition, as most of these proteins are abundant in the uterine fluid, they could also participate in the protection of the reproductive system from pathogen contamination during its formation (**Figure 12**). Once embedded in the eggshell mineral, the exact mechanism by which these occluded molecules could act as antimicrobials is still unknown; however, we hypothesize that they could be released in an active form upon solubilization of the inner calcified layer during embryonic development (**Figure 12**), and potentiate innate immunity in proximity to this biomineralized barrier. In the cuticle, they might also directly protect from pathogens or regulate microbiome composition of the shell surface (see the review by Kulshreshtha et al., 2022 (3)) (**Figure 12**). A common point that emerges from the physicochemical and structural analyses of these twenty proteins is that the majority of them are cationic proteins or have positively charged patch(es) on their surface. This physicochemical property is believed to constitute the hallmark of antimicrobial peptides and proteins (especially those that directly interact with bacteria leading to bacterial growth inhibition or death). For certain proteins (OC-17, VMO1, OCX-32, OVAX, Ex-FABP), 3D structure analysis reveals a clear bipolar distribution of negative and positive charges on their molecular surface. These regions could form hotspots of interaction with ions of the mineral phase (namely calcium or carbonate ions) during eggshell formation, with eggshell proteoglycans, or with yet non-identified protein or non-protein partner(s). In addition, five of these eggshell antimicrobial proteins bind glycosaminoglycan moieties (VMO1, AvBD11, OVAX, MSMB3 and pleiotrophin), which opens new research avenues to explore their interactions with avian eggshell glycosaminoglycans and proteoglycans. From this data, we hypothesize that regulation of the specific crystal size, shape, and orientation is likely to involve a specific orchestration of the eggshell tripartite participants (proteins, glycosaminoglycans, mineral ions) that may differentially interact at specific phases of eggshell formation. Some of these proteins are found in all eggshell layers (eggshell membranes, calcified layer and cuticle: OC-17, OCX-36, TENP, OVAX, cystatin, ovoinhibitor, OCX-32, lysozyme C, and ovotransferrin). In contrast, AvBD9, ovodefensin A1, and MSMB3 have been identified only in the eggshell membranes. VMO1, AvBD11, AvBD10, ovostatin, avidin and Ex-FAPB are present in both eggshell membranes and the calcified layer, while other eggshell AMPPs were identified only in the calcified layer (lysozyme G and pleiotrophin). This review, based on the biochemical and structural analyses of eggshell proteins, provides an original approach to decipher the role of calcifying matrix proteins in the chemical defense of the egg in avian and non-avian species.

## Author Contributions

TM and NG wrote the first draft of the manuscript and integrated the writting contributions of all coauthors (JG, MH, PM, SR-G). All authors reviewed and revised the manuscript prior to submission, and approved the final manuscript content. They warrant that this review manuscript is not under consideration for publication elsewhere.

## Conflict of Interest

The authors declare that the research was conducted in the absence of any commercial or financial relationships that could be construed as a potential conflict of interest.

## Publisher’s Note

All claims expressed in this article are solely those of the authors and do not necessarily represent those of their affiliated organizations, or those of the publisher, the editors and the reviewers. Any product that may be evaluated in this article, or claim that may be made by its manufacturer, is not guaranteed or endorsed by the publisher.

## Abbreviations

ACC, amorphous calcium carbonate; AMPP, antimicrobial peptide and protein; AvBD, avian beta-defensin; BPI, bactericidal permeability increasing; C-ter, carboxy-terminal; CTL, C-type lectin; ESM, eggshell membrane; GAG, glycosaminoglycan; LBP, lipopolysacharide binding protein/; LXN, latexin; NMR, nuclear magnetic resonance; N-ter, amino-terminal; OC, ovocleidin; OCX, ovocalyxin; OvoD, ovodefensin; PDB, protein data bank; TSR, thrombospondin type-1 repeat.
